# Golden Needle Mushroom: A Culinary Medicine with Evidenced-Based Biological Activities and Health Promoting Properties

**DOI:** 10.3389/fphar.2016.00474

**Published:** 2016-12-07

**Authors:** Calyn Tang, Pearl Ching-Xin Hoo, Loh Teng-Hern Tan, Priyia Pusparajah, Tahir Mehmood Khan, Learn-Han Lee, Bey-Hing Goh, Kok-Gan Chan

**Affiliations:** ^1^Biomedical Research Laboratory, Jeffrey Cheah School of Medicine and Health Sciences, Monash University MalaysiaBandar Sunway, Malaysia; ^2^Novel Bacteria and Drug Discovery Research Group, School of Pharmacy, Monash University MalaysiaBandar Sunway, Malaysia; ^3^Department of Pharmacy, Abasyn University PeshawarPeshawar, Pakistan; ^4^Center of Health Outcomes Research and Therapeutic Safety (Cohorts), School of Pharmaceutical Sciences, University of PhayaoPhayao, Thailand; ^5^Division of Genetics and Molecular Biology, Faculty of Science, Institute of Biological Sciences, University of MalayaKuala Lumpur, Malaysia

**Keywords:** *Flammulina velutipes*, enoki, mushroom, nutritional value, biological activity

## Abstract

*Flammulina velutipes* (enoki, velvet shank, golden needle mushroom or winter mushroom), one of the main edible mushrooms on the market, has long been recognized for its nutritional value and delicious taste. In recent decades, research has expanded beyond detailing its nutritional composition and delved into the biological activities and potential health benefits of its constituents. Many bioactive constituents from a range of families have been isolated from different parts of the mushroom, including carbohydrates, protein, lipids, glycoproteins, phenols, and sesquiterpenes. These compounds have been demonstrated to exhibit various biological activities, such as antitumour and anticancer activities, anti-atherosclerotic and thrombosis inhibition activity, antihypertensive and cholesterol lowering effects, anti-aging and antioxidant properties, ability to aid with restoring memory and overcoming learning deficits, anti-inflammatory, immunomodulatory, anti-bacterial, ribosome inactivation and melanosis inhibition. This review aims to consolidate the information concerning the phytochemistry and biological activities of various compounds isolated from *F. velutipes* to demonstrate that this mushroom is not only a great source of nutrients but also possesses tremendous potential in pharmaceutical drug development.

## Introduction

In addition to their nutritional value, folk medicine has long recognized mushrooms for their wide spectrum of therapeutic and prophylactic uses. Many medicinal mushrooms are important ingredients in Traditional Chinese Medicine, such as *Flammulina velutipes* (enokitake), *Lentinus edodes* (shiitake), and *Grifola frondosa* (maitake) (Sullivan et al., 2006). While their properties remained unknown to the scientific community for a long time, in recent decades, there has been significant research focused on the sources, medicinal properties and applications of mushrooms (Encarnacion et al., 2012; Kalač, 2013; Soares et al., 2013).

From a nutritional stand point, these health promoting mushrooms have high nutritional value. They contain dietary fiber, are low in calories, have a high content of protein consisting of all the essential amino acids, minerals and vitamins and are free of cholesterol (Karaman et al., 2010). Beyond their nutritional value, mushrooms have great potential for production of useful metabolites, making them a prolific resource for drug isolation and development (Leung et al., 1997). Mushrooms are now gaining worldwide recognition as a functional food as well as a potential source of nutraceuticals which may reduce severity of, prevent or treat illnesses. The current research on these medicinal mushrooms has, in fact, now progressed beyond validating their traditional medical uses and into the isolation and production of bioactive compounds against specific illnesses (Wang et al., 2012c; Liu et al., 2014).

*F. velutipes* is also commonly known as enokitake, velvet shank or golden needle mushroom winter mushroom. The synonyms for *F. velutipes are Agaricus nigripes, Agaricus velutipes, Collybia eriocephala, Collybia veluticeps, Collybia velutipes, Collybidium velutipes, Gymnopus velutipes, Myxocollybia velutipes, Panaeolus veluticeps, Paxillus veluticeps, Phylloporus veluticeps, and Pleurotus velutipes* (Information was retrieved from MycoBank website http://www.mycobank.org, 1st August 2016). The cultivated F. velutipes has a pure white bean sprout look with a velvety stem topped with a tiny snowy-white cap; while the wild varieties appear in different colors ranging from orange to brown, and have a larger, shiny cap. The significant difference in the appearance of wild and the cultivated *F. velutipes* is attributed to the cultivation of *F. velutipes* without the exposure to light which leads to its white color while the wild ones are brown. *F. velutipes* normally grows on dead elm trees and has been found abundantly on diseased elm trees caused by Dutch elm infection (Ingold, 1980). The species of *Flammulina* have also been reported to occur ubiquitously on a wide variety of deciduous trees such as poplar, plum, maple and birch (Sharma et al., 2009).

*F. velutipes* (Curtis) Singer is one of the most popular edible mushrooms that possesses a wide spectrum of interesting biological activities. It is found ubiquitously throughout the north-temperate regions including North America, Europe and Asia (Ingold, 1980). Historically, it has been has been cultivated for consumption and medicinal use in China since 800 AD. Currently, *F. velutipes* is among the four most widely cultivated mushrooms globally due to its desirable taste, aroma and high nutritional value. It is commonly available in the market or groceries stores sold in vacuum packages. This mushroom is also well known for its curative properties for liver diseases and gastroenteric ulcers (Ingold, 1980).

Research on *F. velutipes* has clearly shown it possesses various pharmacological properties including anticancer, antimicrobial, antioxidant, and immunomodulatory properties, demonstrating that *F. velutipes* has great potential for successful bioprospecting. We aim to give an overview of the present knowledge regarding the bioactive chemical constituents and pharmacological potential of *F. velutipes*.

## Taxonomic classification of *F. velutipes*

**Table:** 

**Taxonomic level**	**Taxonomic name**
Kingdom	Fungi
Phylum	*Basidiomycota*
Class	*Agaricomycetes*
Order	*Agaricales*
Family	*Physalacriaceae*
Genus	*Flammulina*
Species	*F. velutipes*

## Chemical composition and nutritional value of *F. velutipes*

Similar to many other edible mushrooms, *F. velutipes* is consumed as a delicacy, noted for the pleasant aroma and texture it gives to a dish. More importantly, consuming *F. velutipes* can provide key nutrients such as proteins, vitamins, minerals, unsaturated fatty acids and fiber. Additionally, consumption of *F. velutipes* can confer health promoting effects including immunity enhancement, blood cholesterol and blood pressure lowering effects as well as chemopreventive effects by virtue of the bioactive constituents contained in the mushroom which are ingested during consumption. However, it is important to note that the composition of the beneficial compounds present in *F. velutipes* can be highly influenced by the growing site, types of substrate, maturity of the mushroom at the harvesting stage and also the post-harvest handling including the processing and storage conditions. All these factors could account for the variability in composition data published by different studies examining the same mushroom, in addition to the intraspecific genetic variability of mushrooms from different provenance and producers (Reis et al., 2012). We have consolidated the data available in the current literature reporting the nutrient analysis of *F. velutipes* collected from several regions in order to provide further insight into the nutritional benefits of consuming this mushroom.

Based on the findings of Kalač (2013), the dry matter of both wild and cultivated mushrooms is relatively low, usually within the range of 80–140 g/kg. For *F. velutipes*, the dry matter of both wild and cultivated *F. velutipes* are within the normally reported range for mushrooms, measuring between 93 and 114 g/kg (Dikeman et al., 2005; Ko et al., 2007; Beluhan and Ranogajec, 2011; Reis et al., 2012). The low dry matter of the mushrooms accounts for their high water content that leads to shorter shelf life of the fruiting bodies. However, in a comparison among cultivated mushrooms, Dikeman et al. (2005) found that the dry matter of cultivated raw *F. velutipes* was the highest among the other cultivated mushrooms sampled for the study. However, this was not the case for the studies that examined the wild *F. velutipes* collected from European regions (Beluhan and Ranogajec, 2011; Pereira et al., 2012). Furthermore, the dry matter content of *F. velutipes* was reduced after cooking while the dry matter of the other mushrooms was increased after cooking, suggesting that cooking can result in losses for *F. velutipes*.

The data on approximate composition and energy value of both wild and cultivated *F. velutipes* are summarized in Table 1. All the studies reviewed showed that carbohydrates and proteins are the two major constituents contained in the dry matter of *F. velutipes*. No obvious difference is seen in carbohydrate and protein content between the wild and cultivated *F. velutipes*. Given the fact that, similar to other mushrooms, *F. velutipes* has low dry matter and fats content, it is thus a low energy delicacy. Furthermore, the calculated energy value is suggested to be somewhat overestimated as some of the carbohydrates are only partially digestible or indigestible, such as chitin and mannitol (Kalač, 2013). It is interesting to note that a slight difference can be observed between the energy value of wild and the cultivated *F. velutipes*, where the energy value of cultivated *F. velutipes* appears higher than the wild *F. velutipes*, ranging from 410.1 to 459.3 kcal/kg fresh weight and from 342.0 to 398.2 kcal/kg fresh weight, respectively. The energy value was computed according to the following equation: Energy (kcal) = 4 × (g carbohydrate) + 3.75 × (g protein) + 9 × (g fat) (Reis et al., 2012).

**Table 1:** **The proximate composition of ***F. velutipes*** in dry weight basis**.

**Proximate composition (g/kg dry matter)**	**Origin of growing site**		**References**
	**Wild**	**Cultivated**	
Dry matter	93.2–119.5	103.0–114.0	Dikeman et al., 2005; Ko et al., 2007; Beluhan and Ranogajec, 2011; Pereira et al., 2012; Reis et al., 2012
Carbohydrates	426.0–708.5	580.0–871.4	Ko et al., 2007; Beluhan and Ranogajec, 2011; Akata et al., 2012; Pereira et al., 2012; Reis et al., 2012
Proteins	232.0–275.0	178.9–279.5	Dikeman et al., 2005; Ko et al., 2007; Beluhan and Ranogajec, 2011; Akata et al., 2012; Pereira et al., 2012
Fats	17.3–70.0	18.4–73.3	Dikeman et al., 2005; Ko et al., 2007; Beluhan and Ranogajec, 2011; Akata et al., 2012; Pereira et al., 2012; Reis et al., 2012
Ash	72.5–74.0	73.9–104.0	Ko et al., 2007; Beluhan and Ranogajec, 2011; Akata et al., 2012; Pereira et al., 2012; Reis et al., 2012
Energy (kcal/kg fresh weight)	342.0–398.2	410.1–459.3	Ko et al., 2007; Beluhan and Ranogajec, 2011; Pereira et al., 2012; Reis et al., 2012

*Energy (kcal) = 4 × (g carbohydrate) + 3.75 × (g protein) + 9 × (g fat) (Reis et al., 2012).

### Protein and amino acids

Protein represents the most critical component contributing to the nutritional value of a particular food, due to the fact that fats and carbohydrates are rarely lacking in a diet. As one of the major components in the dry matter of *F. velutipes*, protein accounts for approximately 178.9 g/kg dry weight to 279.5 g/kg dry weight of *F. velutipes* (Dikeman et al., 2005; Ko et al., 2007; Beluhan and Ranogajec, 2011; Akata et al., 2012; Pereira et al., 2012). The large range in the protein concentrations could be explained by the fact that protein concentration in this mushroom been shown to vary depending on the growth substrate, size of pileus, time of harvest and the availability of nitrogen sources in the growth substrate. The protein content of *F. velutipes* is comparable to many green leafy vegetables— in the range of 200–300 g/kg dry weight (Singh et al., 2001; Gupta et al., 2005)—demonstrating that *F. velutipes* is a good source of protein. Several unique studies even quantified the concentration of amino acids from *F. velutipes*. Amino acids are the important monomeric building blocks of proteins and can further classified into two classes, the essential and nonessential amino acids. The essential amino acids can only be obtained through dietary intake while the non-essential amino acids are those that can be synthesized by the body. Varying concentrations of free amino acids contained in *F. velutipes* were demonstrated in the studies reviewed (Table 2) (Smiderle et al., 2008; Kim et al., 2009; Beluhan and Ranogajec, 2011). Based on these studies, the major amino acids found in *F. velutipes* were L-glutamic acid, L-alanine, glycine and L-lysine, which contributed 2.6–3.0% of dry weight. Furthermore, essential amino acids such as methionine, valine, isoleucine, leucine, lysine, phenylalanine and threonine were detected in *F. velutipes* (Smiderle et al., 2008; Kim et al., 2009; Beluhan and Ranogajec, 2011). Amino acids also play an important role in contributing to the pleasant taste of mushrooms. For instance, aspartic and glutamic acids are the two amino acids that contribute the monosodium glutamate-like or palatable taste while the alanine, glycine, threonine and serine give a sweet taste to the mushrooms. Beluhan and Ranogajec (2011) found that among the amino acids in *F. velutipes*, amino acids that give MSG-like taste made up the highest composition, followed by sweet, bitter and tasteless amino acids. The study also showed that among a variety of mushrooms, the total content of amino acids that determined the taste characteristics of *F. velutipes* was the lowest at 27.87 mg/g dry weight, accounting for the delicate and very mild in taste of *F. velutipes* unlike other mushroom species which have stronger flavor and taste (Beluhan and Ranogajec, 2011).

**Table 2:** **Concentration of amino acids of ***F. velutipes*****.

**Amino acids (mg/g dry weight)**	**Source of** ***F. velutipes***		
	**Local supermarket, Korea (Kim et al., 2009)**	**Municipal market, Brazil (Smiderle et al., 2008)**	**Forests, Croatia regions (Beluhan and Ranogajec, 2011)**
D,L-O-Phosphoserine	1.31	nt	nt
Taurine	1.74	nt	nt
L-Aspartic acid	2.81	nt	2.59
L-Threonine	6.41	10.047	5.21
L-Serine	6.83	7.686	0.21
L-Glutamic Acid	31.54	9.975	29.98
D,L-α-Aminoadipic Acid	0.64	nt	nt
Glycine	6.13	28.482	0.15
L-Alanine	26.86	7.591	1.95
L-Valine	1.76	6.539	1.54
L-Cysteine	6.32	8.76	1.39
L-Methionine	0.06	3.108	0.03
L-Cystathionine	1.89	nt	nt
L-Isoleucine	0.37	5.09	0.44
L-Tyrosine	0.99	3.471	0.04
L-Phenylalanine	0.19	5.654	1.23
D,L-β-Amino-i-Butyric Acid	2.16	nt	nt
γ-AminoButyric Acid	11.63	nt	nt
Ethanolamine	1.68	nt	nt
D,L & allo-Hydroxylysine	0.3	nt	nt
L-Ornithine	12.55	nt	nt
L-Lysine	6.21	30.896	5.68
L-Histidine	2.44	1.456	2.54
L-Carnosine	0.56	nt	nt
L-Arginine	1.27	3.88	0.49
L-Leucine	0.49	5.404	0.73
Proline	nt	4.947	nt

*nt, not tested*.

Besides that, various bioactive proteins also have been isolated from *F. velutipes*. Fungal immunomodulatory protein, FIP-fve, mainly extracted from fruiting body of *F. velutipes* has been studied extensively and explored for its diverse bioactivities including immunomodulatory, anticancer and anti-inflammatory properties (Chang et al., 2013, 2014; Lee et al., 2013). Ribosome inactivating proteins such as flammin, velin, velutin and flammulin are present in the fruiting bodies and extract of the mushroom (Wang and Ng, 2000, 2001; Ng and Wang, 2004). Studies have also reported isolation of proteins such as hemagglutanin which has mitogenic and antiproliferative functions, ice binding proteins and also the flammutoxin as a cytolysin (Tadjibaeva et al., 2000; Ng et al., 2006; Raymond and Janech, 2009). In addition, proflamin, a glycoprotein enzyme with anticancer activity, and asparaginase were also discovered in the mycelium of the mushroom (Maruyama and Ikekawa, 2007; Eisele et al., 2011; Kotake et al., 2011).

### Carbohydrates

Based on the available data, carbohydrates generally constitute roughly one-half of the total dry weight of *F. velutipes*. Cultivated *F. velutipes* have a slightly higher median value of carbohydrate content compared to the wild *F. velutipes* (Ko et al., 2007; Beluhan and Ranogajec, 2011; Akata et al., 2012; Pereira et al., 2012; Reis et al., 2012). Carbohydrates in *F. velutipes* can be categorized into three main groups: monosaccharides such as ribose, mannose, glucose, xylose, fucose, galactose, and fructose; disaccharides such as sucrose and trehalose, and polysaccharides such as chitin, β-glucan and starch (Dikeman et al., 2005; Kim et al., 2009; Beluhan and Ranogajec, 2011; Pereira et al., 2012; Reis et al., 2012). The concentration of each individual group of carbohydrates contained in *F. velutipes* is tabulated in Table 3. The observed differences in sugar content may be due to geographical factors such as soil conditions, as well as the cultivation method and also the analytical method used. Glucose and trehalose were the two major sugar components detected in both wild and cultivated *F. velutipes* (Dikeman et al., 2005; Kim et al., 2009; Beluhan and Ranogajec, 2011; Pereira et al., 2012; Reis et al., 2012). It was indicated that sugars participate in cellular energy metabolism and also the synthesis of structural polysaccharides of the mushroom (Barros et al., 2008). Moreover, sugars are only a small portion of the total carbohydrate content while the remaining portion consists of other polysaccharides such as starch, chitin and β-glucan.

**Table 3:** **Concentration of monosaccharides, disaccharides and polysaccharides of ***F. velutipes*****.

**Carbohydrates (mg/g dry weight)**		**Provenance**		**References**
		**Wild**	**Cultivated**	
Monosaccharides	Fucose	nt	12	Dikeman et al., 2005
	Mannose	72.3	7.51–61	Dikeman et al., 2005; Kim et al., 2009; Beluhan and Ranogajec, 2011
	Glucose	120.1	11.34–575	Dikeman et al., 2005; Kim et al., 2009; Beluhan and Ranogajec, 2011
	Ribose	nt	11.47	Kim et al., 2009
	Xylose	nt	28–51.56	Dikeman et al., 2005; Kim et al., 2009
	Fructose	nd	379.23	Kim et al., 2009; Reis et al., 2012
	Mannitol	59.8–79.0	79.97	Beluhan and Ranogajec, 2011; Pereira et al., 2012; Reis et al., 2012
	Galactose	nt	27	Dikeman et al., 2005
Disaccharides	Trehalose	29.7–150.8	2.33–216.82	Kim et al., 2009; Beluhan and Ranogajec, 2011; Pereira et al., 2012; Reis et al., 2012
	Sucrose	nt	nd, 7.41	Kim et al., 2009; Reis et al., 2012
Polysaccharides	Chitin	nt	97	Dikeman et al., 2005
	β-glucan	nt	2	Dikeman et al., 2005
	Starch	nt	150	Dikeman et al., 2005

*nd, not detected; nt, not tested*.

Recently, there have been increased investigations into the bioactivities of polysaccharides extracted from both the fruiting body and mycelium of *F. velutipes*. *F. velutipes* derived polysaccharides were found to possess many health promoting properties, such as antioxidant and anticancer activity, immunomodulation, hepatocyte protection and even the ability to treat learning and memory impairment (Pang et al., 2007; He and Zhang, 2013; Wu et al., 2014; Yang et al., 2015) (Zhang et al., 2013). Beta-glucan is one of the many interesting polysaccharides found in *F. velutipes*, having demonstrated anticancer properties (Smiderle et al., 2006).

Carbohydrate content also includes dietary fiber, which is known to be high in mushrooms. According to Kalač (2013), both insoluble and soluble dietary fiber content were shown to make up between 4.2–9.2% and 22.4–31.2% of dry weight in mushrooms. Dikeman et al. (2005) revealed that approximately 29.3% of the dry weight of raw *F. velutipes* consists of dietary fibers; with 90% of the total dietary fiber being insoluble while the remaining 10% is soluble. Furthermore, it was found that the total dietary fiber content increased as a result of cooking for *F. velutipes* (Dikeman et al., 2005). A previous study also demonstrated that the high dietary fiber content of *F. velutipes* extract conferred hypolipidemic effect, lowering total cholesterol levels in animals on a high fat diet (Yeh et al., 2014).

### Lipids and fatty acids

Generally, mushrooms are a low energy delicacy with a caloric value of approximately 350 kcal per kg owing to its low dry matter and lipid content (Kalač, 2013). The lipid content of *F. velutipes* falls in the range of 17.3–73.3 g/kg dry weight and is composed of sterols, sphingolipids and fats (Ko et al., 2007; Beluhan and Ranogajec, 2011; Akata et al., 2012; Pereira et al., 2012). The total fatty acid content (in terms of dry weight basis) of *F. velutipes* consists predominantly of monounsaturated and polyunsaturated fatty acids (79.23%) while saturated fatty acids make up the remaining 20.67% (Günç Ergönül et al., 2013). Table 4 summarizes the percentages of the individual fatty acids detected in *F. velutipes*. Linoleic acid is the major fatty acid contained in *F. velutipes*, making up 40.93–56.33% of the total fatty acid content. Linoleic acid is an essential fatty acid for mammals and is the precursor for biosynthesis of many important inflammatory mediators such as arachidonic acid and prostaglandins in mammals (Salem et al., 1999). Thus, *F. velutipes* may represent an important food source for humans or other animals to obtain sufficient amounts of the essential fatty acids our body requires but which cannot be synthesized. Many studies also demonstrated that sterols extracted from *F. velutipes* possess antiproliferative activity against several cancer cell lines and have the potential to be developed as chemotherapeutic agents (Yi et al., 2012, 2013a); while mycosterol, derived from the extract of *F. velutipes*, exhibits potent hypolipidemic activity (Yeh et al., 2014).

**Table 4:** **Main fatty acids (percentage) found in ***F. velutipes*****.

**Main fatty acids**	**Percentage (%)**	**References**
Stearic acid	1.38–3.56	Pereira et al., 2012; Günç Ergönül et al., 2013
Palmitic acid	10.31–14.56	Pereira et al., 2012; Günç Ergönül et al., 2013
Oleic acid	15.08–16.43	Pereira et al., 2012; Günç Ergönül et al., 2013
Linoleic acid	40.93–56.33	Pereira et al., 2012; Günç Ergönül et al., 2013

### Micronutrients

The ash content in mushrooms generally ranges within 5–12% of dry matter and 72.5–104.0 g/kg of ash was reported in *F. velutipes* (Ko et al., 2007; Beluhan and Ranogajec, 2011; Akata et al., 2012; Pereira et al., 2012). The concentration of elements contained in *F. velutipes* are tabulated in Table 5. Similar to other mushrooms (Manzi et al., 1999; Smiderle et al., 2008; Zeng et al., 2012), potassium is the most abundant mineral element contained in *F. velutipes* (28.00–28.98mg/g dry weight), followed by phosphorus (8.80–9.40 mg/g dry weight). This indicates that a 100g portion of *F. velutipes* can contribute to around 9% of the recommended daily intake of potassium which is 3100 mg/day according to FDA (Akhter et al., 2003). The high potassium and low sodium content may also make *F. velutipes* of potential benefit in a salt restricted diet for suitable for those with hypertension or heart disease. In fact, studies also suggested that potassium from fruit and vegetables can reduce blood pressure (John et al., 2002; He et al., 2006). The other minerals present in minor amounts include copper, iron, zinc and sulfur, which are also important supplementary elements in our diet (Smiderle et al., 2008). It is also interesting to note that the wild Australian *F. velutipes* contain higher copper and potassium levels as compared to other Australian mushrooms species (Zeng et al., 2012).

**Table 5:** **Content of major mineral elements in ***F. velutipes*****.

**Minerals**	**Concentration (mg/g dry weight)**	**References**
Calcium	0.36–1.18	Smiderle et al., 2008; Zeng et al., 2012
Potassium	28.00–28.98	Smiderle et al., 2008; Zeng et al., 2012
Sodium	0.657–0.755	Smiderle et al., 2008; Zeng et al., 2012
Magnesium	0.68–1.43	Smiderle et al., 2008; Zeng et al., 2012
Zinc	0.048–0.068	Smiderle et al., 2008; Zeng et al., 2012
Selenium	<0.00050	Smiderle et al., 2008
Lithium	<0.00020	Smiderle et al., 2008
Copper	0.057	Zeng et al., 2012
Manganese	0.0096	Smiderle et al., 2008
Iron	0.0963	Smiderle et al., 2008
Sulfur	6.06	Zeng et al., 2012
Phosphorus	8.80–9.40	Smiderle et al., 2008

It has been demonstrated that the vitamin content in mushrooms are species and source-dependent (Pereira et al., 2012; Nakalembe et al., 2015). According to Pereira et al. (2012), *F. velutipes* was shown to contain tocopherols (α-tocopherol, β-tocopherol and δ-tocopherol, but not γ-tocopherol) (0.6 μg/g dry weight), ascorbic acid (238 μg/g dry weight), β-carotene (3.4 μg/g dry weight) and lycopene (0.2 μg/g dry weight). These vitamins or micronutrients may play an important role in contributing to the mushroom's antioxidant activity (Breene, 1990; Pereira et al., 2012).

### Health promoting constituents/non-nutrients

**Figure 1:**
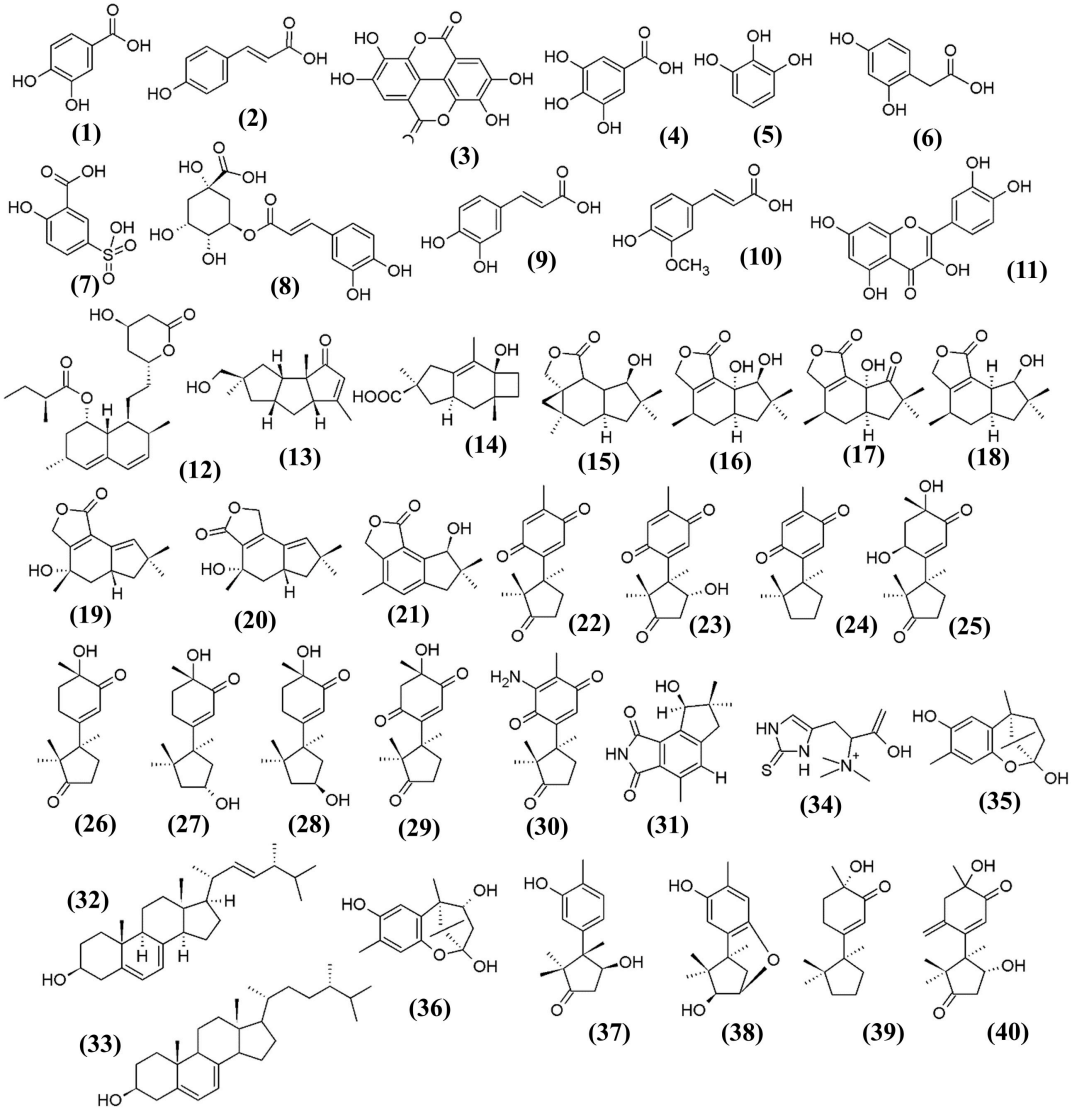
**The chemical structures of bioactive constituents isolated from ***F. velutipes*****.

Besides the macronutrients and micronutrients present in *F. velutipes*, phenolics, especially the phenolic acids which are known to be the main antioxidants in the mushroom, have been detected (Kim et al., 2008; Rahman et al., 2015). Phenolics are heterocyclic compounds and well-recognized for their potent antioxidant activity; they are particularly effective in protecting the body against diseases associated with oxidative stresses such as cancer, cardiovascular disease and diabetes. Rahman et al. (2015) identified several polyphenolic antioxidants present in the methanol:dichloromethane fraction of *F. velutipes*, including the protocatechuic acid **(1)**, *p*-coumaric **(2)**, and ellagic acid **(3)**. These bioactive phenolic and polyphenolic compounds confer the antioxidative effect of *F. velutipes* in preventing the progression of atherosclerosis (Rahman et al., 2015). Gallic acid **(4)**, pyrogallol **(5)**, homogentisic acid **(6**), 5-sulfosalicylic acid **(7)**, chlorogenic acid **(8)**, caffeic acid **(9)**, ferulic acid **(10)**, and quercetin **(11)** are other examples of phenolic compounds detected in *F. velutipes*, ranging from 9.0 to 26.0 μg/g dry weight (Kim et al., 2008). The chemical structures of these bioactive compounds are illustrated in Figure 1. Interestingly, an interspecies comparison study by Zeng et al. (2012) showed that *F. velutipes* had the highest phenolic content of 2.823 ± 0.007 mg gallic acid equivalent (GAE)/g extract among other mushrooms harvested from Australia. The study also suggested the variation in the phenolic content in those mushrooms may be ascribed to the different geographical locations and also dependent on the ability of a particular sub-species in the synthesis of phenolic compounds (Zeng et al., 2012).

In addition, various novel sesquiterpenes and norsequiterpenes were also identified from the extract of *F. velutipes*. These compounds exhibited several bioactivities such as anticancer, antibacterial and antioxidant activity (Wang et al., 2012a,d). Lovastatin, **(12)** which is effective in lowering cholesterol levels, was detected in the fruiting bodies of *F. velutipes*, estimated at 90.8 ± 2.0 mg/kg dry weight (Chen et al., 2012).

In summary, *F. velutipes* has excellent nutritional content—high protein and carbohydrate content, low fat content and also high polyunsaturated fatty acid content, making it an excellent food suitable for low calorie diet. However, as noted, there are differences in the chemical composition between the cultivated and the wild samples of *F. velutipes*, including carbohydrates, proteins and fatty acid profiles. Based on the current literature, it should be noted that data on digestibility and bioavailability of *F. velutipes* or indeed any other mushrooms are still lacking. In future, more studies could explore the bioavailability of specific nutrients or bioactive constituents contained in *F. velutipes* as well as investigate the changes in the individual constituents during different preservation methods, storage conditions and different cooking processes.

## Biological activities of *F. velutipes*

**Figure 2:**
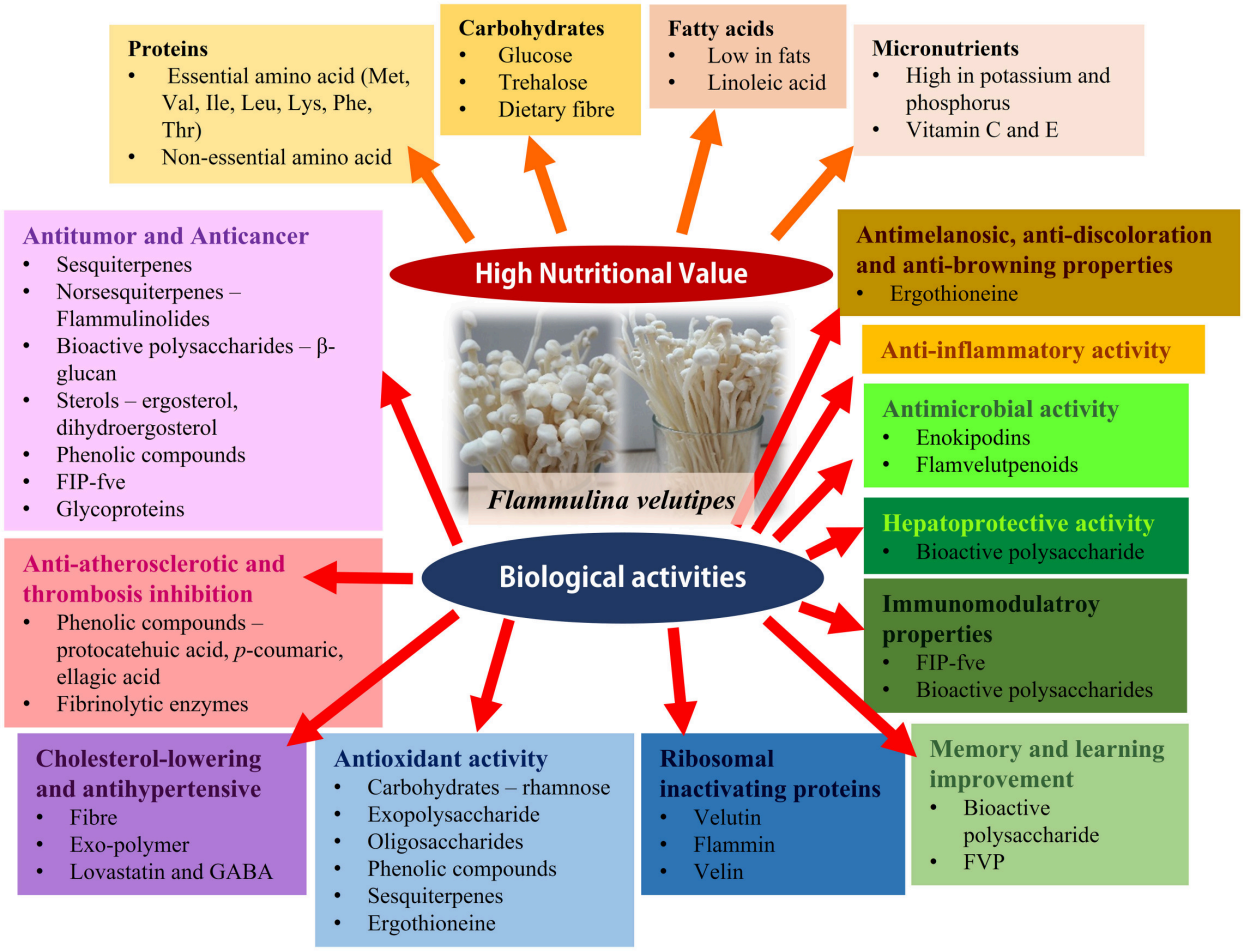
**The graphical abstract of the nutritional values and biological activities of ***F. velutipes*****.

*F. velutipes* has been reported to have multiple beneficial effects on human health. They include antitumour, anticancer and anti-atherosclerotic activity, thrombosis inhibition, antihypertensive and cholesterol lowering effects, anti-aging and antioxidant properties, ability to restore neurotransmitters associated with memory and learning, anti-inflammatory, immunomodulatory and anti-bacterial activities (Gu and Leonard, 2006; Karaman et al., 2010; Lee et al., 2013; Wu et al., 2014; Rahman et al., 2015; Yang et al., 2015). These biological activities of *F. velutipes* are summarized in Table 6; a summary of the biological activities of *F. velutipes* in a graphical from is depicted in Figure 2.

**Table 6:** **The bioactivities of extracts and constituents isolated from ***F. velutipes*****.

**Bioactivities**	**Tested substance**	**Experimental design**	**Dosage and results**	**References**
Antitumor and anticancer	Water extract of *F. velutipes*	*In vitro* cell culture, BT-20, MCF-7 and MDA-MB-231 cells	Growth inhibition against BT-20, MCF-7 and MDA-MB-231 at IC_50_ = 30, 150, and 75 μg/mL, respectively	Gu and Leonard, 2006
	Flammulinolide A **(15)** Flammulinolide B **(16)** Flammulinolide C **(17)** Flammulinolide F **(20)**	*In vitro* cell culture, Hela, HepG2 and KB cells	Flammulinolide A **(15)**, B **(16)** and F **(20)**—cytotoxicity against KB cell at IC_50_ = 3.9, 3.6 and 4.7 μM Flammulinolide C **(17)**—cytotoxicity against Hela at IC_50_ = 3.0 μM	Wang et al., 2012c
	Enokipodin B **(22)** Enokipodin D **(23)** Enokipodin J **(30)** 2,5-cuparadiene-1,4-dione **(24)**	*In vitro* cell culture, HepG2, MCF-7, A549 and SGC7901 cells	Moderate cytotoxicity against all the tested cancer cell lines with IC_50_ within 20 to 100 μg /mL	Wang et al., 2012d
	A novel norsequiterpene alkaloid **(31)**	*In vitro* cell culture, KB cell	Cytotoxicity against KB cells at IC_50_ = 16.6 μM	Xu et al., 2013
	Alkaline-soluble polysaccharide from cell wall of *F. velutipes*	SC-180 mouse model	Significant inhibition (94.1–97.8%) on the growing of SC-180 tumor at dose of 15 mg/kg	Leung et al., 1997
	Hot-water extract of polysaccharides from *F. velutipes* fruiting body	S-180 mice tumor model and SMMC-7721 human hepatoma cells (*in vitro*)	Inhibition (54.7–58.1%) on proliferation of S-180 implanted tumor *in vivo* at dose of 24 mg/kg 59.5% inhibition on proliferation of SMMC-7721 cells at 50 mg/*F. velutipes* polysaccharide *in vitro*	Jiang et al., 1999
	Polysaccharide extracts from *F. velutipes* mycelium	*In vitro* cell culture, BEL-7402 cell	FvP-2 and FvP 3 showed inhibition against BEL-7402 for 45% and 40% at 20 μg/mL and 40 μg/mL, respectively	Zhao et al., 2013
	Triple helical polysaccharide	*In vitro* cell culture, BCG-823 and A549	FVP-1 showed inhibitory rate of 32.3% against A549 at 100 μg/mL; 78% against BGC-823 at 200 μg/mL FVP-2 showed inhibitory rate of 27.9% against A549 at 200 μg/mL; 95% against BGC-823 at 200 μg/mL	Yang et al., 2012
	Sterols (ergosterol **(32)** (54.8%) and 22, 23-dihydroergosterol **(33)** (27.9%)) extracted from *F. velutipes*	*In vitro* cell culture, SGC, HepG2, A549 and U251	Cytotoxicity against SGC cell with IC_50_ at 11.99 μg/mL; HepG-2 cell with IC_50_ at 9.3 μg/mL; A549 lung cancer cell with IC_50_ at 20.4 μg/mL and U251 glioblastoma with IC_50_ at 23.42 μg/mL	Yi et al., 2012, 2013a,b
	FIP-fve, an immunomodulatory protein	*In vitro* cell culture, A549 cell BNL hepatoma-bearing female BALB/c mice	Reduced expression of RACGAP1 gene responsible for metastasis Reduced tumor size of BNL hepatoma in mice (10 mg/kg oral administration) by expression of IFN-γ through ERK/MAPK signaling pathway; inhibited angiogenesis through CD4^+^T-cell-derived IFN-γ Inhibited the proliferation of A549 mediated through the increased p53 and p21 expression Acted as adjuvant with HPV-16 E7, increased lifespan of tumor-bearing mice through increased HPV-16 E7-specific antibodies and T cells	Chang et al., 2010, 2013
	Proflamin, a glycoprotein extracted from mycelium of *F. velutipes*	Tumor bearing mice model with B-16 melanoma and Ca755 adenocarcinoma	Increased mean survival time of mice with B-16 and Ca-755 for 86 and 84%, respectively, at 10 mg/kg administrated orally	Ikekawa et al., 1985
Anti-atherosclerotic activity	Methanol:dichloromethane fraction of *F. velutipes* (contained protocatechuic acid **(1)**, *p*-coumaric **(2)** acid and ellagic acid **(3)**	*In vitro* lag time of conjugated diene formation in LDL molecule and TBARS inhibition assay	Prolonged the lag time of CD formation up to 120 min at 1 μg/mL concentration Inhibited 48.71% of TBARS formation at 1 mg/mL concentration	Rahman et al., 2015
Thrombosis inhibitory activity	Fermented extract of *F. velutipes*	Coagulability test, thrombin time Fibrinolytic activity test	Prolonged thrombin clotting time for 358.6 ± 0.4 s (2.2 × control) Demonstrated fibrinolytic activity on fibrin plate with clear zone of 20 ± 0.5 mm^2^	Okamura et al., 2001
	Fibrinolytic enzyme isolated from culture supernatant of *F. velutipes*	Fibrinolytic and fibrinogenolytic assays (SDS-PAGE analysis)	Hydrolyzed fibrin α-chain followed by the *γ-γ* chain and β-chain (similar hydrolysis pattern as of plasmin) Hydrolyzed Aα- and Bβ- chains of fibrinogen	Park et al., 2007
Cholesterol-lowering effect and antihypertensive activity	*F. velutipes* powder (FVP) and water extract (FVE)	Male Syrian hamsters under high cholesterol diet (lipoproteins determination)	Reduced levels of serum TC (28.7%), TG (33.6%), LDL (54.5%) and LDL/HDL ration (61.8%) and also liver TC (37.6%) and TG (46.1%) of high cholesterol diet hamsters supplemented with 3% of FVP Reduced levels of serum TC (27.0%), TG (28.6%), LDL (48.5%) and LDL/HDL ration (57.9%) and also liver TC and TG of high cholesterol diet hamsters supplemented with 3% of FVE	Yeh et al., 2014
	Exopolymer extracted from mycelium culture	Sprague-Dawley male rat under hyperlipidemic diet	Reduced the plasma triglyceride (20.2%), TC (21.7%), LDL (27.6%) and liver TC (21.4%) in hyperlipidemic rat at 100 g/kg body weight for 4 weeks	Yang et al., 2002
	*F. velutipes* fiber	Male F344/DuCrj rats	Reduced levels of serum TC (9.93%), and VLDL+IDL+LDL (20.6%) when fed with 50 g/kg for 4 weeks as compared to control fed with cellulose diet only	Fukushima et al., 2001
	GABA (6–7%) produced by *F. velutipes*	Spontaneously hypertensive rats	Lowered the systolic blood pressure by ~30 mmHg at 0.9 mg GABA/kg	Harada et al., 2011
	Mycelium culture of *F. velutipes*	*In vitro* ACE inhibitory assay	Showed strong ACE inhibitory effect from 40.7 to 52.8% with IC_50_ from 7.4 to 22.6 mg/mL compared to other basidiomycetes cultures	Kim et al., 2002
Memory and learning improvement	Polysaccharides extracted from *F. velutipes*	Scopolamine-induced memory and learning impaired rat model	Increased SOD and GSH-Px activities in the brain; inhibited TBARS formation in the brain from 100 to 400 μg/kg Restored the neurotransmitters (DA, NE, 5-HT and Ach) level in the brain of scopolamine treated rat Increased the expression of *p*-CaMK II and connexin 36 in the brain	Yang et al., 2015
	Water extract of *F. velutipes* and polysaccharides extracted from the water extract with ultrasonic	*In vitro* AChE inhibitory activity	Water extract demonstrated ~ 20% AChE inhibitory effect The FVP extracted using ultrasonic displayed AChE inhibitory rate of 18.51% at 0.6mg/ml	Yang et al., 2011
Ribosome inactivating protein	Velutin	ELISA kit—glycohydrolase inhibitory activity assay Cell-free rabbit reticulocyte lysate system	At 5 mg/mL, velutin showed inhibition against HIV-1 reverse transcriptase, α-glucosidase, β-glucosidase and β-glucuronidase for 102.3, 8.3, 62.3 and 64.7%, respectively Cell-free translation-inhibitory activity (prevented protein synthesis of ^3^H-leucine at IC_50_ of 0.29 nM)	Wang and Ng, 2001
	Flammin	Cell-free rabbit reticulocyte lysate system	Inhibited translation at IC_50_ of 1.4 nM	Ng and Wang, 2004
	Velin	Cell-free rabbit reticulocyte lysate system	Inhibited translation at IC_50_ of 2.5 nM	Ng and Wang, 2004
	Flammulina	Cell-free rabbit reticulocyte lysate system	Inhibited ^3^H-leucine incorporation into protein at IC_50_ of 0.25 nM	Wang and Ng, 2000
Antioxidant activity	Intracellular polysaccharides from *F. velutipes* mycelium SF-08	*In vitro* antioxidant assays–DPPH, superoxide, hydroxyl and reducing power assays *In vivo* antioxidant status–SOD, GSH-Px and LPO assays	DPPH scavenging activity reached up to 66.38% at 1 μg/mL Superoxide scavenging activity reached up to 84.29% at 1 μg/mL Hydroxyl radical scavenging activity reached up to 84.42% at 1 μg/mL Reducing power reached up to 1.56 at 1 μg/mL Increased SOD and GSH-Px activities and decreased LPO level in all heart, kidney and blood at 800 mg/kg body weight of mice administrated orally	Ma et al., 2015b
	Exopolysaccharide extracted from culture of *F. velutipes* SF-06	*In vitro* antioxidant assays—DPPH, superoxide, hydroxyl and reducing power assays *In vivo* antioxidant status—catalase and TBARS assays	DPPH scavenging activity reached up to 64.53% at 1 μg/mL Superoxide scavenging activity reached up to 67.64% at 1 μg/mL Hydroxyl radical scavenging activity reached up to ~80.0% at 1 μg/mL Reducing power reached up to 1.45 at 1 μg/mL Increased catalase activity and decreased TBARS formation in all heart, kidney and blood at 800 mg/kg body weight of mice administrated orally	Ma et al., 2015a
	Oligosaccharides from *F. velutipes*	*In vitro* antioxidant assays—hydroxyl scavenging and reducing power assays	Hydroxyl scavenging activity reached up to 80.24% at 100 μg/mL Reducing power reached up to 0.856 at 100 μg/mL	Xia, 2015
	Water-soluble polysaccharide	*In vitro* antioxidant assays—superoxide radical scavenging, hydroxyl radical scavenging and reducing power assays	Superoxide radical scavenging activity with an IC_50_ of ~10 mg/mL Hydroxyl scavenging activity with an IC_50_ of ~12 mg/mL Demonstrated reducing power of 1.04 at 5 mg/mL	Wu et al., 2014
	Polysaccharide extracted from mycelia of *F. velutipes*	*In vitro* antioxidant assays—DPPH scavenging and hydroxyl radical scavenging activities	DPPH scavenging activity reached up to 65.58% at 200 μg/mL Hydroxyl scavenging activity reached up to 71.24% at 71.24 μg/mL	Zhao et al., 2013
	Methanol extract of *F. velutipes*	Total phenolic content, *in vitro* antioxidant assays—Trolox equivalent antioxidant capacity, ferric reducing power and ferrous ion chelating activities	Contained 2.823 mg GAE/g extract, highest among the three other mushrooms species Exhibited 221 μmol TE/g extract, 138 μmol Fe[II]-E/g extract and 524 μmol EDTA-E/g extract	Zeng et al., 2012
	Water soluble nucleotide extract from *F. velutipes*	*In vitro* antioxidant assays—ABTS scavenging and total reducing ability	Total reducing power of ~0.3 at 20 mg/mL Exhibited ABTS scavenging rate of ~40% at 20 mg/mL	Cheng et al., 2012
	Enokipodin B **(22)** Enokipodin D **(23)** Enokipodin J **(30)** 2,5-cuparadiene-1,4-dione **(24)**	*In vitro* antioxidant assay—DPPH scavenging activity	Enokipodin J **(30)** with IC_50_ at 78.6 μM; 2,5-cuparadiene-1,4-dione **(24)** with IC_50_ at 80.7 μM; Enokipodin B **(22)** with IC_50_ at 154.2 μM and Enokipodin D **(23)** with IC_50_ at 116.5 μM	Wang et al., 2012d
Immunomodulatory properties	FIP-fve purified from extract of *F. velutipes*	*In vitro* experiment—cell proliferation assay murine splenocytes; cytokine profiling (ELISA) *In vivo* experiment—food allergy murine model (oral sensitization by ovalbumin)	Induced proliferation of total murine splenocytes and only the T cells that is APC-dependent (co-cultured with irradiated splenocytes) (10–40 μg/mL) Resulted Th1-skewed immune response (prominent IFN-γ; littler or no IL-4 production in the supernatant) (10–40 μg/mL) Resulted increased serum IFN-γ in mice fed with 100–400 μg per mouse every day for 8 days Reduced systemic anaphylaxis-like symptom score and plasma histamine level in OVA challenged mice treated with FIP-fve	Hsieh et al., 2003
	FIP-fve purified from extract of *F. velutipes*	*In vitro* experiment—cell cycle analysis, cytokine analysis and Western blot	Induced G1/G0 to S phase proliferation in PBMC at 100 μg after 72 h Increased IFN-γ production dose-dependently in PBMC until reached plateau at 100 μg/mL Induced ICAM-1 expression dose-dependently; activated p38 MAP kinase (increased expression of phospho-p38)	Wang et al., 2004
	FIP-fve purified from extract of *F. velutipes*	*In vitro* experiment—Ca^2+^ analysis, Western blot and RT-PCR	Caused elevation of intracellular Ca^2+^ concentration release in PBMC (increase transiently at 30 s after treated with FIP-fve and reached maximum at 70 s) Maximum activation of PKC-α at 3 h incubation with 7.69 μM concentration Increased expression of IFN-γ mRNA upon treatment with FIP-fve at7.69 μM for 48 h	Ou et al., 2009
	FIP-fve	*In vitro* experiment—eosinophil survival under IL-5 (apoptosis analysis), apoptotic protein expression (flow cytometry), RT-PCR	Reduced the protective effect of IL-5 for eosinophils from apoptosis (at 10 and 25 μg/mL), increased in early apoptotic and late apoptotic eosinophils Diminished the anti-apoptotic Bcl-2 in eosinophil stimulated with IL-5 when treated with FIP-fve Downregulated IL-5Rα proteins and mRNA expression on eosinophils	Hsieh et al., 2007
	FIP-fve purified from extract of *F. velutipes*	*In vivo* experiment—mouse model of allergic asthma (sensitization to OVA intraperitoneally 1st to 14th day, followed by intranasal challenge 3% OVA)	Both pre-treatment and post-treatment of FIP-fve suppressed airway hyperactivity (assessed with whole-body barometric plethysmography) Reduced IgE (54.7–58.6%) and increased IgG2a (83.2–144.5%) in the serum Decreased Th2 cytokines (IL-4, IL-5 and IL-13 and IL-10), increased Th1 and Treg cytokines (IFN-γ and TGF-β) in the serum Reduced inflammation and epithelial cell thickness (histological examination)	Lee et al., 2013
	FIP-fve purified from extract of *F. velutipes*	*In vivo* experiment—mouse models of allergic asthma (sensitization to HDM or DM intraperitoneally 1st to 14th day, followed by intranasal challenge 50 μg HDM/DM)	Reduced airway hyperresponsiveness (assessed with whole-body barometric plethysmography) Reduced infiltrating cells (eosinophils and lymphocytes) in mice challenged by HDM or DM upon treatment with FIP-fve At humoral level, increased IgG2a while decreased IgE in the serum Reduced Th2 cytokines while increased Th1 and Treg cytokines in serum and BALF Reduced inflammation and epithelial cell thickness (histological examination)	Chang et al., 2015; Chu et al., 2015
	FIP-fve purified from extract of *F. velutipes*	Female BALB/c mice with RSV intranasal challenge (2 × 10^5^PFU) *In vitro* experiment—plaque formation assay (HEp-2 cell), RT-PCR	FIP-fve administrated orally (2 days before to 6 days after RSV infection)—decreased airway hyperreactivity Reduced IL-6 while increased IFN-γ Reduced RSV infection rate in lung tissues Inhibited plaque formation (from 111.0 plaque count to 17.3 and 0 at 7.5 μM and 30 Mm, respectively Decreased IL-6 mRNA expression	Chang et al., 2014
	FIP-fve purified from extract of *F. velutipes*	*In vivo* experiment—neutropenia mouse model induced by docetaxel	10 mg/kg mouse for 3 days (oral gavage) before docetaxel injection—restored docetaxal-induced myelotoxicity (elevated hemoglobin level), protected the bone marrow and haematopoietic cells from damages by docetaxel, prevented damages on the bone microenvironment Partial protection for intestinal villi against docetaxel-induced interstinal injuries	Ou et al., 2015
	Polysaccharide purified from *F. velutipes* mycelium	*In vitro* experiment—NO production assay, TNF-α production assay and IL-1 ELISA	Enhanced NO, TNF-α, IL-1 production by macrophages in a dose dependent manner	Yin et al., 2010
	Polysaccharide derived from *F. velutipes* mycorrhizae (PFVM)	*In vivo* experiment—oral gavage of PFVM twice a day for 60 days on female Wister mice, weight ratio of thymus and spleen, flow cytometry, cytokine profile *In vitro* experiment—isolation of mice spleen lymphocytes, MTT	Increased the weight ratio of thymus and spleen at doses of 2 g/kg and 4 g/kg, respectively Increased the percentage of CD3^+^ and CD4^+^thymocytes dose-dependently in the peripheral blood, thymus, spleen of the mice Increased IL-2 and TNF-α in the serum dose-dependently Increased the percentage of CD3^+^, CD4^+^ and ratio of CD4^+^ /CD8^+^ in spleen lymphocytes	Yan et al., 2014
	Water-soluble polysaccharide from *F. velutipes*	*In vitro* experiment—NO production assay, IL-1, IL-6 and TNF-α ELISA and lymphocyte proliferation assay	Increased the NO production from macrophages dose-dependently from 5 to 160 μg/mL Increased production of IL-1β at 20 μg/mL while increased production of IL-6 and TNF-α at 5 μg/mL Promoted lymphocytes proliferation from 50 to 500 μg/mL in dose-dependent manner	Wu et al., 2014
	Water extract of *F. velutipes*	*In vitro* experiment—proliferation assay, cytokine profile	Increased proliferation of splenocytes at concentrations ranging from 10 to 1000 μg/mL Increased Th1 cytokine productions	Ryu et al., 2014
	Boiling water extract of *F. velutipes*	*In vitro* experiment—cytokine profile (ELISA), cytotoxicity assay	Stimulated the production of IFN-γ from large intestinal lamina propria leukocytes Promoted the cytotoxicity of large intestinal lamina propria leukocytes isolated from rat against YAC-1 cells	Lee et al., 2011
Melanosis inhibitory activity	Ergothioneine **(34)** containing hot water extract of *F. velutipes*	*In vitro* experiment—mushroom polyphenol oxidase inhibition assay, RT-PCR *In vivo* experiment—Immersion treatment, postharvest melanosis	Inhibited mushroom polyphenol oxidase activity by 58% after 300 s at 0.38 mg ergothioneine/mL extract, and in a dose-dependent manner Lowered expression of proPO gene in group fed with *F. velutipes* extract Suppressed the polyphenol oxidase activity in the hemolymphs of the organisms supplemented with *F. velutipes* extract diet (25% in wet diet each day for 7 days) 0.5% *F. velutipes* extract in immersing solution prevented melanosis in the carapace during ice storage	Encarnacion et al., 2010, 2011a,b; Encarnacion et al., 2012
	Ergothioneine **(34)** containing hot water extract of *F. velutipes*	*In vitro* experiment—total lipid hydroperoxides analysis and metmyoglobin formation	Suppressed metMB formation and HPO accumulation by 1 mL of extract (10 g fresh material to 100 g of minced tuna meat) Showed anti-discoloration activity with 1 mL of extract (10 g fresh material to 100 g of minced tuna meat)	Bao et al., 2010b
	Ethyl acetate of *F. velutipes* mycelium grown in 2% glucose	*In vitro* experiment—anti-tyrosinase activity assay	Demonstrated inhibitory activity of 58.8% against tyrosinase at 0.5 mg/mL of extract	Kim et al., 2011
Antimicrobial activity	Methanol and chloroform extracts of *F. velutipes*	*In vitro* experiment—plate diffusion	Methanol extract exhibited strongest antibacterial activity against *S. aureus* from horse wound infection with 19.75 mm zone inhibition Chloroform extract exhibited strongest antibacterial activity against *Bacillus* sp. from animal skin with 15.75 mm zone inhibition	Karaman et al., 2010
	Methanol extract of *F. velutipes*	*In vitro* experiment—microdilution method	Displayed antibacterial activity against *B. subtilis* ATCC6633, *B. pumilus* NCTC8241, *S. aureus* ATCC6538 and *P. aeruginosa* ATCC9027 with MIC measured at 12.5, 3.125, 50 and 50 mg/mL, respectively	Nedelkoska et al., 2013
	*F. velutipes* colony	*In vitro* experiment—duel culture technique	Showed complete replacement against *Ophiostoma ulmi* Showed partial replacement against *F. oxysporum, P. funereal* and *F. culmirum* LM2091	Borhani et al., 2011
	Enokipodin A **(35)** Enokipodin C **(36)**	*In vitro* experiment—plate diffusion and broth dilution	Enokipodin A **(35)**—against *B. subtilis* LMA0011 (21–30 mm), *B. subtilis* IFO12734 (21–30) mm and *S. aureus* AHU1142 (16–20 mm) at 50 μg Enokipodin A **(35)** showed antibacterial activity against *B. subtilis* LMA0011 with MID of 3.12 μg Enokipodin C **(36)**—against *B. subtilis* LMA0011 (16–20 mm), *B. subtilis* IFO12734 (16–20 mm) and *S. aureus* AHU1142 (16–20 mm) at 50 μg Enokipodin C **(36)** showed antibacterial activity against *B. subtilis* LMA0011 with MID of 6.25 μg	Ishikawa et al., 2001, 2005
	Flamvelutpenoid A–D **(37 to 40)**	*In vitro* experiment—broth dilution	Displayed weak activity against *E. coli, B. subtilis* and methicillin-resistant *S. aerues* with MIC of >100 μM	Wang et al., 2012b
	Enokipodin F **(26)** Emokipodin G **(27)** Enokipodin I **(29)** Enokipodin J **(30)** 2,5-cuparadiene-1,4-dione **(24)** Enokipodin B **(22)** Enokipodin D **(23)**	*In vitro* experiment—microdilution technique	Enokipodins I **(29)**, J **(30)**, B **(22)**, D **(23)** and 2,5-cuparadiene-1,4-dione **(24)** showed activity against *B. subtilis* with IC_50_ measured at 164.3, 151.2, 140.5, 167.6 and 154.6, respectively Enokipodin F **(26)**, G **(27)** and I **(29)** showed activity against *A. fumigatus* with IC_50_ measured at 229.1, 233.4 and 235.1, respectively	Wang et al., 2012d
Anti-inflammatory activity	*F. velutipes* in dried powder form	*In vitro* experiment—Griess assay and ELISA (TNF-α)	Exhibited NO inhibitory activity of IC_50_ at 24 μg/mL Exhibited TNF-α inhibitory activity of IC_50_ at 99 μg/mL	Gunawardena et al., 2014
	Water extract of *F. velutipes*	*In vitro* experiment—NO inhibition assay and Western blot	Inhibited NO production Inhibited LPS-induced iNOS and COX-2 expression in macrophages	Kang, 2012
Hepatoprotective activity	Water-soluble polysaccharide of *F. velutipes* (FVP2)	*In vitro* experiment—viability test, ALT analysis	Promoted proliferation of primary culture of mouse hepatocytes at concentrations ranging from 25 to 200 μg/mL Reduced the intracellular release of ALT from hepatocytes induced by CCl_4_ intoxication	Pang et al., 2007

### Antitumor and anticancer

With the increasing rate of life-threatening neoplastic diseases in recent years, development of more effective antitumor drugs is a research area of great relevance (Ajith and Janardhanan, 2007). Currently, chemotherapeutic agents used in cancer treatment are able to slow down the progress of the disease; however, they are also toxic toward healthy, non-neoplastic cells (Tan et al., 2016b). An alternative approach currently being explored intensively is pursuing anticancer agents from natural food products to inhibit the onset of cancer (Chung et al., 2010; Chan et al., 2012; Goh et al., 2014; Tan et al., 2016a). Over the years, bioactive compounds derived from *Inonotus obliquus* (chaga mushroom) and *L. edodes* (shiitake) have been shown to exhibit anticancer effects against certain cell lines such as human leukemic U937 cells, stomach adenocarcinoma AGS cells, lung carcinoma A549 cells, sarcoma S-180 cells and human colorectal adenocarcinoma HT-29 cells. (Ou et al., 2005; Chung et al., 2010; Jeff et al., 2013). *F. velutipes* has also been shown to contain bioactive compounds with antitumor and anticancer properties (Gu and Leonard, 2006; Smiderle et al., 2006; Wang et al., 2012b; Yi et al., 2013b).

In 2003, Ikekawa (2001) presented an epidemiological study spanning 15 years (1972–1986) which showed that the cancer related death rates of farmers who grew *F. velutipes* mushroom—assuming that they would have eaten some of the mushrooms they farmed—were lower by 39% when compared to comparable populations not involved in mushroom farming (Monro, 2003). Experimental evidence also demonstrated that *F. velutipes* extract possesses anticancer properties, and several anticancer compounds have been isolated from *F. velutipes* in recent decades. Gu and Leonard (2006), among others, revealed the anticancer potential of fruiting bodies extract from *F. velutipes*, which was particularly effective against breast cancer cell lines. In the study, *F. velutipes* extract was shown to inhibit the growth of both estrogen-receptor positive (ER+) MCF-7 and estrogen-receptor negative (ER−) MDA-MB-231 human breast cancer cell lines. Furthermore, the extract induced apoptosis in the breast cancer cells and also caused 99% inhibition of colony formation of MCF-7 (Gu and Leonard, 2006).

Recently, several novel bioactive compounds with anticancer activities were isolated from the cultures of *F. velutipes*. A research group from China discovered a sesquiterpene with a novel carbon skeleton, known as flammulinol A **(13)**, together with sterpuric acid **(14)**, an isolactarane sesquiterpene and six isolactarane-related norsesquiterpenes (flammulinolides A–G) (**15** to **21**) from the solid culture of *F. velutipes* (Wang et al., 2012c). These isolactarane-related norsesquiterpenes **(15** to **17, 19** to **21)** extracted from the solid culture of *F. velutipes* were found to possess cytotoxic effect against several cancer cell lines. Flammulinolide A (**15**) showed cytotoxic effect against KB cells (human nasopharyngeal carcinoma) and HepG2 (human hepatocellular liver carcinoma) with an IC_50_ of 3.6 and 34.7 μM, respectively, whereas flammulinolide C (**17**) exhibited strong cytotoxicity against HeLa cells (human cervical adenocarcinoma) with an IC_50_ of 3.0 μM (Wang et al., 2012c). Other groups have reported the isolation of sesquiterpenes (**22** to **30**) from solid culture of *F. velutipes* grown on cooked rice, sesquiterpenes **(22** to **24, 30)** were reported to have moderate cytotoxicity against human tumor cell lines: HepG2 (liver cancer cells), MCF-7 (breast cancer cells), SGC7901 (stomach cancer cells), and A549 (lung cancer cells) with IC_50_ within 20 to 100 μg /mL (Wang et al., 2012d). A new nonsesquiterpene alkaloid **(31)** derived from fermented rice substrate of *F. velutipes* was also discovered to exhibit inhibitory effect against human KB cells *in vitro* (Xu et al., 2013).

Aside from the sesquiterpenes, bioactive polysaccharides from *F. velutipes* are also potential anticancer agents. The polysaccharides of *F. velutipes* demonstrated antitumor and anticancer properties against sarcoma SC-180 mouse model and S-180 mice tumor model *in vivo* and hepatoma SMMC-7721 cells *in vitro* (Leung et al., 1997; Jiang et al., 1999). Recently, crude polysaccharide from mycelia of the mushroom was also found to reduce the proliferation of human BEL-7402 lung cancer cells by 45% at 640 μg/mL (Zhao et al., 2013). Triple helix structured polysaccharides extracted by ultrasonic wave from *F. velutipes* exhibited high inhibitory effect against BGC-82 gastric cancer cells, suggesting a potential role in prevention of gastric carcinoma (Yang et al., 2012). Beta-glucan, which is well known for its antitumor activity (Zhang et al., 2007; Mantovani et al., 2008), was also isolated from *F. velutipes* (Smiderle et al., 2006).

Sterols extracted from *F. velutipes*, consisting of mainly ergosterol **(32)** (54.8%) and 22, 23-dihydroergosterol **(33)** (27.9%), were found to be potential therapeutic agents against stomach, liver, lung cancer and gliomas. It showed potent antiproliferative activity against human SGC cells (stomach cancer cells) with an IC_50_ of 11.99 μg/mL, HepG-2 cells with IC_50_ at 9.3 μg/mL, A549 lung cancer cell with IC_50_ at 20.4 μg/mL and U251 glioblastoma with IC_50_ at 23.42 μg/mL (Yi et al., 2012, 2013a,b). Studies have also been done to improve the bioavailability, biodistribution and solubility of sterols from *F. velutipes* through encapsulation in liposomes, mixed micelles nanoformulation and microemulsion (Yi et al., 2012, 2013a,b).

*F. velutipes* extract was found to contain phenolic compounds such as protocatechuic acid **(1)**, *p*-coumaric **(2)**, and ellagic acid **(3)** (Rahman et al., 2015) which have anticancer effects (Ferguson et al., 2005; Seeram et al., 2005). Both *p*-coumaric **(2)** and ellagic acid **(3)** have potent antioxidative effect on human colon cells, HT-29 and HCT 16 cell lines, respectively (Ferguson et al., 2005; Seeram et al., 2005). Ellagic acid was shown to be able to reduce hepatic P450 level and also its catalytic activities *in vitro*, thus decreasing the metabolism of carcinogens that can cause chemically induced cancer (Ahn et al., 1996).

The biological activities of FIP-fve, a bioactive protein isolated from the mushroom *F. velutipes*, have also been investigated extensively. FIP-fve belongs to a fungal immunomodulatory protein (FIP) family that modulates immune responses, including antitumor activity (Chang et al., 2013). Chang et al. (2010) suggested that FIP-fve is able to reduce the expression of RACGAP1 gene and also reduce its reporter activity (Chang et al., 2013). The RACGAP1 gene is responsible for the survival and metastatic activity of lung cancer cells, thus, silencing of this gene reduces the migration of cancer cells (Wang et al., 2011). Besides that, FIP-fve also increased tumor suppressor gene p53 expression and also its downstream gene, p21, thus attenuating the proliferation of A549 lung cancer cells (Chang et al., 2013). In another study, Chang et al. (2010) demonstrated that the oral administration of FIP-fve reduced the tumor size of BNL hepatoma-bearing mice and suggested that the antitumor effect was mediated by IFN-γ-induced tumor growth inhibition effect involving both innate and specific immunity and ERK/MAPK signaling pathway. The study also showed that the antitumor effect of FIP-fve was mediated through the inhibition of angiogenesis by CD4^+^ T-cell-derived IFN-γ. In addition, the study showed that the expression of MHC class I and II and co-stimulatory molecule CD80 on peripheral blood mononuclear cell was also increased, suggesting the oral administration of FIP-fve exerted antitumor effect through upregulation of presenting ability of APCs (Chang et al., 2010). These findings are in concordance with another *in vivo* study whereby FIP-fve protein activated the maturation of splenic dendritic cells, an APC and stimulated antigen-specific CD8^+^ T-cell immune responses. Administration of FIP-fve as adjuvant therapy along with HPV-16 E7 vaccine to tumor bearing mice resulted in increased production of HPV-16 E7-specific antibodies and increased expansion of HPV-16 E7-specific interferon (IFN)-γ-producing CD4^+^ and CD8^+^ T cells compared with HPV-16 E7 vaccine alone, resulting prolonged lifespan of the mice (Ding et al., 2009). Overall, the evidence is strongly suggestive that FIP-fve is a potential agent for the development of novel adjuvants for cancer immunotherapy.

Glycoproteins found in the fruiting body and mycelium of *F. velutipes* also exhibit anticancer effect (Zhang et al., 2007). Proflamin, an acidic glycoprotein isolated from the mycelium enhanced several immunosuppression processes and exhibited antiproliferative effects against various cancer cells. It was demonstrated to be able to prolong the lifespan of mice bearing B-16 melanoma and adenocarcinoma 755 tumor cells (Ikekawa et al., 1985). When given as combination therapy along with vaccines or surgery, it also inhibited the growth of sarcoma S-180, L1210 leukemia ascite cells and Meth-A fibrosarcoma. The study also revealed another protein-bound polysaccharide, EA6 significantly inhibited the proliferation of Meth-A fibrosarcoma. Similar to FIP-fve, the antitumor effects of EA6 which are manifested by virtue of strengthening the specific and innate immunity, were shown to be mediated by CD4^+^ T cells (Maruyama and Ikekawa, 2007). To date, it is obvious that many bioactive compounds with effective anticancer and antitumor properties have been isolated from different parts of *F. velutipes*, indicating that this mushroom is a good source for future development of chemotherapeutic agents. Nevertheless, studies are still required to further study the bioavailability of these bioactive compounds derived from *F. velutipes* and also their exact mechanisms of action.

### Vascular diseases: anti-atherosclerotic and thrombosis inhibition

Oxidized low density lipoprotein (ox-LDL) has long been known to be the key player in the early events of the atherosclerosis cascade. Lipid hydroperoxides are formed when polyunsaturated fatty acid is oxidized by free radicals, and the continuous oxidation and reduction of the hydroperoxides will further augment the peroxidation process. Recently, Rahman et al. (2015) demonstrated the polyphenolic compounds present in methanol:dichloromethane (M:DCM) fraction of *F. velutipes* are able to retard LDL oxidation, thus possibly being effective in impeding the progression of atherosclerosis. Protocatechuic acid **(1)**, *p*-coumaric **(2)** and ellagic acid **(3)** were identified from the M:DCM fraction of *F. velutipes* that showed the longest lag time of conjugated diene formation and inhibition of TBARS formation (Rahman et al., 2015). The study suggested that the inhibition of the peroxidation processes may be attributed to the chain breaking actions of the phenolic compounds which are able to reduce the alkoxyl or peroxyl radicals to alkoxides or hydroperoxides, respectively, interfering with the peroxidation process (Rice-Evans et al., 1997). In addition, the study also proposed that the antiatherosclerotic effect demonstrated by the M:DCM fraction of *F. velutipes* may be mediated through anti-inflammatory effects. Protocatechuic acid **(1)** is known to prevent the adhesion of monocytes to tumor necrosis factor-alpha(TNF-α)activated endothelial cells, leading to a reduction of the expression of vascular cell adhesion molecule 1 (VCAM-1), intercellular adhesion molecule 1 (ICAM-1) and also nuclear factor kappa-light-chain-enhancer of activated B cells (NF-κB) binding activity, thus reducing the formation of atheroma (Kakkar and Bais, 2014). Another phenolic constituent of the fraction, *p*-coumaric acid **(2)** is able to inhibit ADP-induced platelet aggregation, interrupt the arachidonic acid cascade and decrease thromboxane B2 and lipopolysaccharide-induced prostaglandin E2 production, hence inhibiting the formation of plaque and inflammation process (Luceri et al., 2007). In conclusion, the phenolic components in *F. velutipes*, which have high ability toward withstanding the oxidation of LDL as well as their anti-inflammatory activity, may be the agents contributing to the overall anti-atherosclerotic properties of the extract.

Thrombosis is one of the important initial events that takes place during accelerated atherosclerosis (Walters et al., 1994). One study revealed that the use of *F. velutipes* in fermentation may produce fermented products which possess preventive activity against thrombosis. It has been demonstrated that the addition of *F. velutipes* in fermentation process resulted in end-product with a prolonged thrombin clotting time (358.6 ± 0.4 sec) of 2.2-fold than that of the control. Further experimentation has also demonstrated a strong fibrinolytic activity on a fibrin plate (Okamura et al., 2001). A more recent study successfully purified and characterized a fibrinolytic enzyme from the culture supernatant of *F. velutipes* mycelium (Park et al., 2007). The fibrinolytic enzyme (FVP-I), a protease from *F. velutipes* was shown to be a direct-acting fibrinolytic and fibrinogenolytic agent which elicits direct cleavage of fibrin and fibrinogen without the need of plasminogen activator, thus demonstrating potential as thrombolytic therapy (Park et al., 2007). Collectively, the evidence suggests that *F. velutipes* is a potential source of bioactive substances for drug development in vascular disease prevention.

### Antihypertensive and cholesterol-lowering effect

Almost 17 million deaths, approximately 1/3 of the total deaths in a year are due to cardiovascular diseases; and among these, complications of hypertension account for approximately 9.4 million deaths. Hypertension, together with other health risk factors, is associated with highly detrimental complications including increased probability of heart attack, stroke and kidney disease as well as other chronic diseases. These risk factors include tobacco use, diabetes and hypercholesterolemia (World Health Organization, 2013). The consumption of herbs and mushrooms as dietary supplement has been known to be beneficial to people with cardiovascular diseases (Tan et al., 2016c). Owing to the rich content of dietary fiber, mushrooms can help in cholesterol metabolism and absorption, thereby lowering the risk of cardiovascular diseases. There is also a growing interest in *F. velutipes* as an attractive source of various biologically active components including dietary fiber, polysaccharides and mycosterol that have been long known to possess cholesterol and blood pressure lowering effects (Yeh et al., 2014). In fact, *F. velutipes* was shown to contain the highest fiber content as compared to other mushrooms such as *L. edodes*, oyster cap fungi and cap fungi (Yang et al., 2001).

Yeh et al. (2014) investigated the effect of the active components of both *F. velutipes* powder and *F. velutipes* extract on the lipid metabolism of male hamsters on a high fat diet. The study revealed that both *F. velutipes* extract and powder at dose of 3% are capable of reducing the level of TC (total cholesterol), TG (triacylglycerol), LDL (low density lipoprotein cholesterol), and LDL/HDL (high density lipoprotein cholesterol) in the serum and liver of the hamsters significantly (Yeh et al., 2014). This is in agreement with the findings of another study whereby the exo-polymer of *F. velutipes* exerted hypolipidemic effect on diet-induced hyperlipidemic rats. Significant reduction in plasma triglyceride, plasma TC, LDL and liver TC levels were observed from the animals administered *F. velutipes* exo-polymer at 100g/kg body weight for 4 weeks (Yang et al., 2002). It was also demonstrated that the *F. velutipes* fiber diet resulted in reduction of plasma TC and increased fecal cholesterol excretion and liver LDL receptor mRNA level in rats (Fukushima et al., 2001).

Mushrooms are known to elicit hypocholesterolemia and also to possess anti-hypertensive properties. Chen et al. (2012) reported the detection of both lovastatin **(12)** and γ-aminobutyric acid (GABA) in the fruiting body of *F. velutipes*. Lovastatin **(12)** is used clinically for its inhibitory effect on cholesterol production, thereby reducing the risk of coronary heart disease. Many studies have shown that food products containing GABA are able to lower the blood pressure of hypertensive subjects (Aoki et al., 2003; Inoue et al., 2003; Hayakawa et al., 2004). Another study reported that the administration of GABA enriched *F. velutipes* powder (0.9mg GABA /kg) was successful in reducing the systolic blood pressure by 30 mmHg in spontaneously hypertensive rats. Of particular note, the study also reported that those with normal blood pressure were not affected by the powder (Harada et al., 2011). These results were suggested to be due to GABA's effects on inhibiting noradrenaline release from the sympathetic nervous system, ameliorating the rise in blood pressure (Hayakawa et al., 2002).

Furthermore, an optimized culture broth used for the growth of *F. velutipes* mycelium was shown to display prominent inhibition of angiotensin converting enzyme with IC_50_ of 22.6 mg/mL (Kim et al., 2002). The authors also highlighted that the use of the culture broth of *F. velutipes* as a source for ACE inhibitor provides many benefits for the future development of anti-hypertensive agents that are important for the treatment for cardiovascular diseases (Kim et al., 2002).

### Memory and learning improvement

Alzheimer's disease is a progressive neurodegenerative disorder characterized by the deterioration of cognition and memory. Studies have indicated that loss of basal forebrain cholinergic neurons involved in learning and memory processes constitutes a pathological hallmark of Alzheimer's disease (Martorana et al., 2010). Besides the cholinergic hypothesis, neurodegenerative disease, including Alzheimer's disease, are also associated with oxidative damage in the brain resulting from an imbalance between reactive oxygen species generation and antioxidant enzyme activity (Wong et al., 2012; Sayyad et al., 2016; Ser et al., 2016). Previous reports revealed that several mushrooms have been demonstrated to exhibit cognitive enhancing activity including *Hericium erinaceus* (Yamabushitake) (Mori et al., 2009), *Inonotus obliquus* (Chaga) (Giridharan et al., 2011) and *Cordyceps militaris* (Tsai et al., 2015). Several recent studies have also sought to investigate the beneficial effects of *F. velutipes* in cognitive function improvement (Yang et al., 2011, 2015).

An *in vivo* study showed that polysaccharides from *F. velutipes* (FVP) were effective against the progression of scopolamine induced learning and memory deficits in rats (Yang et al., 2015). The study revealed that the administration of FVP prevented the reduction of the antioxidant defense enzymes activities and elevation of TBARS levels caused by scopolamine in the rats, showing that FVP improved the memory deficits in the rats through amelioration of oxidative stress (Gao et al., 2012; Yang et al., 2015). FVP was also found to restore the level of the neurotransmitter acetylcholine (ACh) in the hippocampus and cerebral cortex by modulating the activities of its synthetic enzyme, choline acetyltransferase (ChAT) and its hydrolysing enzyme, acetylcholinesterase (AChE). It also normalized the levels of other neurotransmitters including the serotonin, dopamine, and norepinephrine, thereby reversing the effect of scopolamine on the reduction of the neurotransmitters (Yang et al., 2015). These neurotransmitters are known to be involved in both memory and learning functions (Wang et al., 2013). In addition, FVP was also shown to prevent learning and memory impairment by regulating the expression of protein kinases, CaMK II and connexin 36 which plays a role in the synthesis and secretion of neurotransmitters (Yang et al., 2015). Another study also demonstrated that the FVP extracted using ultrasonic methods displayed AChE inhibitory rate of 18.51% at 0.6 mg/ml, suggesting FVP has potential in improvement of learning and cognitive ability (Yang et al., 2011).

### Ribosome inactivating protein

Ribosome inactivating protein (RIP) is well known for exhibiting diverse bioactivities including antitumour, immunomodulatory, abortifacient, and anti-human immunodeficiency (anti –HIV) virus actions (Ng et al., 1992). Wang and Ng (2001) isolated an RIP designated as velutin from *F. velutipes*. Velutin was shown to possess N-terminal sequence which resembles most other plant RIPs to a certain extent, however, its 10kDA molecular weight is much lower compared to the others, which mostly range from 25 to 30 kDA (Ng et al., 1992; Wang and Ng, 2001). Velutin was reported to inhibit the activity of HIV virus reverse transcriptase and also glycohydrolase, mainly α- and β-glucosidases which play a part in HIV infection (Wang and Ng, 2001). Besides that, velutin was also non-teratogenic when tested on mouse embryos (Ng et al., 2010).

Flammin and velin were the other two RIPs discovered from *F. velutipes* (Ng and Wang, 2004). Both of these RIPs do not show much resemblance in terms of N-terminal sequence to the published mushrooms RIPs, instead they show more similarities with angiosperm RIPs. Flammin and velin do not exhibit any RNase and protease activities. This lack of ribonuclease activity further confirms that the cell free translation-inhibitory activity is attributed to ribosome inactivation and not the result of hydrolysis of RNA and protein (Ng and Wang, 2004). Flammulina was another RIP found in the mushroom, and similar to flammin and velin, this protein is also found to be devoid of ribonuclease activity (Wang and Ng, 2000).

### Antioxidant activities

Reactive oxygen species (ROS) refers to free radicals derived from oxygen, including superoxide anion, nitric oxide, hydroxyl radical and hydroxyl peroxide. ROS have important functions such as signaling and regulating the fundamental cellular processes of development such as cell death, oogenesis, spermatogenesis, angiogenesis and redox regulation of cells (Chan et al., 2015). However, excessive accumulation of ROS leads to conditions of oxidative stress which causes damage to lipid, DNA and protein, resulting in detrimental effects to the body (Covarrubias et al., 2008). Antioxidants are substances which can delay, prevent or reverse oxidative damage and are known to prevent several chronic diseases such as cancer and diabetes (Karaman et al., 2010; Lau et al., 2015). Butylated hydroxyanisole and butylated hydroxytoluene are examples of potent synthetic antioxidants, however, some of them are found to be toxic and carcinogenic to human body, thus efforts are now focusing on discovering natural antioxidant products (Moghadamtousi et al., 2014; Ma et al., 2015b; Tan et al., 2015a; Ser et al., 2016). Due to its various biological activities including the antioxidant properties, *F. velutipes*, as one of the most popular edible mushrooms, has attracted a considerable amount of attention in different fields including biochemistry and pharmacology (He and Zhang, 2013).

Over the years, many studies have reported that carbohydrate content, mainly the polysaccharides of *F. velutipes* exhibit antioxidant activity (Ma et al., 2015b; Xia, 2015). In recent decades, studies have reported high antioxidant activities shown by the different carbohydrate products derived from *F. velutipes* by using various extraction methods. Ma et al. (2015b) investigated the antioxidant capacity of the intracellular polysaccharides (IPS) extracted from *F. velutipes* mycelia. The study indicated rhamnose was the major component in IPS responsible for the strong antioxidant activity *in vitro* including the ability to scavenge hydroxyl and DPPH radicals (Ma et al., 2015b). Besides, the study also showed IPS exhibited anti-aging potential in which the anti-aging enzyme, superoxide dismutase (SOD) in the blood, heart and kidney was increased following treatment with increasing concentrations of IPS (Ma et al., 2015b). SOD protects cells from being damaged by superoxide anion radicals by converting them to hydrogen peroxide, a less active free radical (Siu et al., 2013). In another study, exopolysaccharides (EPS) from *F. velutipes* were purified and characterized and it was found that the purified fractions were mainly composed of rhamnose (Ma et al., 2015a). Similarly, these EPS fractions were shown to exhibit potent antioxidant activity *in vitro* such as reducing power and the scavenging capability of hydroxyl, DPPH and superoxide anions (Ma et al., 2015a). This study also demonstrated the purified EPS fractions stimulated anti-aging activity in mice as evidenced by the increased catalase level and decreased malondialdehye content in the organs of the mice. At 800 mg/kg weight, which was the highest dosage of EPS used in the experiment, catalase activity was the highest in heart with 10.12 ± 0.05 U/mg protein. At 800 mg/kg dosage of EPS, the MDA content was the least in liver, 0.62 ± 0.03 nmol/mg protein which is 110% lower than the model control group treated with saline and d-galactose.

Oligosaccharides derived from *F. velutipes* by hydrolysis using hydrogen peroxide also showed a strong hydroxyl radical scavenging of 80.24% at the concentration of 100 μg/mL (Xia, 2015). Wu et al. (2014) extracted polysaccharide, FVP 1-A from *F. velutipes*, which exhibited superoxide radical scavenging ability with an IC_50_ value of about 10 mg/mL, hydroxyl radical scavenging ability with an IC_50_ value of about 12 mg/mL and reducing power of 1.04, showing a high antioxidant capacity. At 200 μg/mL, polysaccharide from liquid culture mycelia extracted using double distilled water, displayed DPPH scavenging rate of 65.85% and hydroxyl radical scavenging rate of 71.24% (Zhao et al., 2013).

Variation in extraction methods may also influence the antioxidant properties of the polysaccharides derived from *F. velutipes*. A study showed that the polysaccharides of *F. velutipes* extracted using various extraction methods (conventional solvent extraction, ultrasound-assisted extraction (UAE), microwave-assisted extraction (MAE), enzymatic aqueous extraction (EAE)) were found to exhibit differential antioxidant activities in different *in vitro* assays. Crude polysaccharides (CFP) demonstrated the highest antioxidant activity in terms of reducing power, EAE polysaccharides (EFP) had the highest hydroxyl radical scavenging and metal chelating activity, whereas UAE polysaccharides (UFP) had the highest DPPH scavenging activity. The researchers deduced that the molecular weight and chemical structure of the polysaccharides obtained from different extraction methods played a role in determining their antioxidant properties (Zhang et al., 2013). UFP and MFP exhibited very much greater DPPH scavenging ability compared to EFP and CFP; the high antioxidant capacity observed may be due to the further alteration of chemical structures and decomposition of polysaccharides caused by the ultrasonic and microwave treatments (Yang et al., 2008).

Aside from polysaccharides, the phenolic compounds are also major naturally occurring antioxidant compounds in mushrooms (Barros et al., 2007; Kim et al., 2008). The total phenolic content in the mushrooms has a positive correlation with its antioxidant property measured by *in vitro* assays such as DPPH assay, hydroxyl assay and lipid peroxidation assay (Karaman et al., 2010). Zeng et al. (2012) demonstrated that *F. velutipes* possesses the highest phenolic content based on it having the highest antioxidant activities in terms of ferric reducing antioxidant power and ferrous ion chelating activity among three other Australian mushrooms. In contrast, Karaman et al. (2009) found that *F. velutipes* has poorer phenolic content compared to other lignicolous fungi, suggesting that the prominent antioxidant activity may due to other secondary biomolecules which had yet to be identified at the time of the study. Besides the fruiting body of *F. velutipes*, the spent culture medium of the mushroom was also shown to contain high amount of phenolic acids and show potent antioxidative action against lipid oxidation, demonstrating its role as a potential antioxidative agent (Bao et al., 2010a). For instance, *p*-coumaric acid **(2)**, one of the hydroxycinnamic acids found in the mushroom, was shown to increase the activity of SOD and inhibited oxidative stress, resulting in reduction of cardiac apoptosis in isoprenol-induced myocardial infarction in rats (Stanely Mainzen Prince and Roy, 2013). Ellagic acid **(3)**, which was detected in the fruiting bodies of *F. velutipes*, and is also present in other fruits and nuts such as pomegranate and walnuts, was shown to be a potent antioxidant exhibiting DPPH radical and hydroxyl radical scavenging, reducing capacity and metal chelating activities (Kilic et al., 2014).

Besides phenols and carbohydrates, a few other bioactive compounds present in *F. velutipes* were shown to exhibit antioxidant activity. Nucleotides derived from *F. velutipes* were reported to possess mild antioxidant activity. They showed reducing power value of 0.5 at 40 mg/mL and ABTS radical scavenging ability of 60% at 50 mg/mL (Cheng et al., 2012). *F. velutipes* was reported to contain 46 mg/100g dry matter of vitamin C which is a well-known antioxidant (Breene, 1990; Fu et al., 2002). Sesquiterpenes **(22** to **24, 30)** extracted from solid culture *F. velutipes* grown on cooked rice were demonstrated to have DPPH radical scavenging ability (Wang et al., 2012d).

Recently, a study investigated the effect of selenium on the antioxidant activity of *F. velutipes* (Milovanovic et al., 2015). Selenium participates in the process of selenoproteins and selenoenzyme synthesis which work to protect cells from free radicals. The study demonstrated that selenium supplemented mycelium had enhanced total phenol content and also enhanced DPPH radicals scavenging ability of the mushroom extract (Milovanovic et al., 2015).

Interestingly, a study conducted by Zhang et al. (2013) demonstrated that the different varieties of *F. velutipes* possess different degrees of antioxidant activities. The investigation included four *F. velutipes* varieties: Fxuexiu (snowy white), FD (off-white), F3415 (yellow) and FYehuang (snuff color). Fxuexiu and F3415 had higher total phenolic and ergothioneine **(34)** (ESH) content compared to the other two. In term of their antioxidant activities, F3415 exhibited the strongest DPPH radical scavenging ability and metal chelating ability, Fxuexiu showed the greatest reducing capability and FD had the most potent hydroxyl radical scavenging ability. Meanwhile, Fyehuang displayed the weakest antioxidant activity among the three other varieties (Zhang et al., 2013). A linear relationship was observed between the phenolic acid and ESH content of the samples and their DPPH radical scavenging ability and reducing power. These findings were concordant with those of Bao et al. (2009) which stated that high DPPH radical scavenging activity of the mushroom was mainly attributed to its ESH content (Bao et al., 2009; Zhang et al., 2013). Furthermore, greater ESH level was associated with a stronger delay in autoxidation of oxymyoglobin (Bao et al., 2010a; Chen et al., 2012).

Based on these studies, it can be concluded that the strong antioxidant activity of *F. velutipes* is attributed to its various bioactive components, namely polysaccharides, phenols, rhamnose sugar, ESH, vitamin C and nucleotides. Different parts of *F. velutipes* including fruiting body, mycelium and even its spent culture medium are sources of potential antioxidants. Different extraction methods of polysaccharides yield extracts with varying antioxidant properties. Antioxidant ability of the mushroom extract also can be increased with the addition of selenium in the medium. Lastly, antioxidant capacity of *F. velutipes* varies between different varieties.

### Immunomodulatory properties: anti-allergy, anti-viral and anti-complement activity

Recently, many studies have been done on medicinal plants in search of compounds that exhibit immunomodulatory properties as it has been discovered that the modulation of the immune system helps to prevent diseases. The currently available chemical drugs used as immunomodulators have shown to possess a higher risk profile when compared to natural immunomodulators (El Enshasy and Hatti-Kaul, 2013; Shukla et al., 2014). Mushrooms have been known for their medicinal value for decades, and many of the compounds extracted from mushrooms have been demonstrated to have modulatory effects on the immune system (Lee et al., 2011). *F. velutipes* is one of the medicinal mushrooms that exhibits immunomodulatory activities. Fungal Immunomodulating Protein (FIP) is one of the main compounds exhibiting immunomodulatory properties in *F. velutipes*. There was also evidence in a recombinant study expressing the recombinant FIP-fve cloned in the expression cassette vector pQE-30 in *E. coli* M15. The recombinant FIP-fve was shown to modulate different cytokine gene expression in mouse spleen cells, including the increased expression of IL-2, IL-4, IFN-γ, TNF-α, LT, and IL-2R (Li et al., 2011).

One of the earliest studies showed that oral administration of FIP extracted from *F. velutipes* (FIP-fve) induced Th-1 predominant allergen-specific immune response and protected the mice from anaphylaxis-like symptoms induced by oral challenge with ovalbumin (OVA) (Hsieh et al., 2003). Therefore, Hsieh et al. (2003) proposed that FIP-fve can be developed as an immunoprophylactic agent against allergic diseases, showing potential to be used clinically in children for food allergy prevention. In the following year, it was found that FIP-fve exerted mitogenic effect on human peripheral blood lymphocytes, acting as a potent activator for lymphocyte proliferation (Wang et al., 2004). The study demonstrated that the activated lymphocytes showed enhanced secretion of interferon-γ (IFN-γ) associated with ICAM-1 expression, both *in vitro* and *in vivo*. Furthermore, the study showed that the expression of IFN-γ in Th1 cells was regulated by p38 MAP kinase pathway in response to FIP-fve (Wang et al., 2004). In addition, Ou et al. (2009) revealed the IFN-γ production induced by FIP-fve in human peripheral mononuclear cells was also mediated by the Ca^2+^ release and PKC-α activation.

These findings were then followed by several studies investigating the anti-allergic effect of FIP-fve against allergen-induced airway diseases (Hsieh et al., 2007; Lee et al., 2013). Hsieh et al. (2007) investigated the effect of FIP-fve on the survival of eosinophils isolated and purified from allergic asthmatic patients in the presence of IL-5. The study indicated that FIP-fve induced apoptosis of eosinophils in the presence of IL-5 (which is a survival factor of eosinophils), thereby preventing eosinophils from undergoing necrosis. Eosinophils are known to be related to allergic diseases, and apoptosis of eosinophils, without the release of their contents, is an important feature in the resolution of inflammation. Furthermore, the study further clarified that the inhibition of the IL-5-mediated survival of eosinophils by FIP-fve was mediated through the upregulation of CD95 expression and the downregulation of BcL-xL and pro-caspase 3 expression (Hsieh et al., 2007). Lee et al. (2013) conducted an *in vivo* study that utilized an OVA-induced chronic airway inflammation murine model to evaluate the effect of FIP-fve against allergic airway diseases. The study indicated that both pre-treatment and post-treatment with FIP-fve successfully suppressed airway inflammation and hyperresponsiveness. FIP-fve was shown to inhibit inflammatory cell infiltration and Th2 cytokines, further strengthening the view that FIP-fve possesses potential as a therapeutic agent for allergy related diseases (Lee et al., 2013). Recent studies also demonstrated the anti-allergic effect of FIP-fve against different allergen-induced airway inflammation in mice (Chang et al., 2015; Chu et al., 2015). Chu et al. (2015) showed oral administration of FIP-fve inhibited house dust mite (HDM)-induced asthma inflammation in mouse model via the modulation of Th1 cytokine production. Meanwhile, Chang et al. (2015) showed that intranasal application of FIP-fve reduced *Dermatophagoides microceras* (DM)-induced airway hyper-responsiveness, airway inflammation and cytokine expression in mice.

Aside from the immunomodulatory properties against allergic diseases, Chang et al. (2014) showed that FIP-fve can be potentially used for viral prevention and therapy. The study revealed that FIP-fve suppressed airway hyperresponsiveness and inflammation as the result of the downregulated IL-6 expression in mice infected by respiratory syncytial virus. Chang et al. (2014) also indicated that pre-treatment of FIP-fve did not prevent RSV infection but inhibited the replication of RSV through reduction in NF-κB translocation and increased IFN-γ expression.

Furthermore, FIP-fve was shown to have the potential to serve as a novel therapeutic protective agent in preventing adverse effects of certain drugs (Ou et al., 2015). This study demonstrated that FIP-fve reversed several side effects of docetaxel—an anticancer drug—against non-small cell lung cancer, without affecting the potency of docetaxel (Ou et al., 2015). FIP-fve was shown to significantly reduce the adverse effects caused by docetaxel with fewer empty vacuoles in bone marrow, less small intestinal mucosa damage and decreased reduction of white blood cell counts in mice. Therefore, the study indicated that FIP-fve showed protective effects against docetaxel-induced bone and intestinal damages and also enhanced WBC counts via induction of G-CSF and IL-20 gene expressions (Ou et al., 2015).

Apart from fungal immunomodulating protein, the polysaccharides extracted from *F. velutipes* are also known to exhibit immunomodulatory properties. Yin et al. (2010) isolated polysaccharides from *F. velutipes* mycelium which were shown to increase nitric oxide (NO) production, IL-1 production and TNF-α production from macrophages in a dose-dependent manner. Another study showed that polysaccharides from *F. velutipes* mycorrhizae (PFVM) increased the body weight of mice and the weight ratio of the thymus and spleen (Yan et al., 2014). According to the study, the T cell subpopulation of thymocytes and splenocytes were modulated by the administration of PFVM; CD3^+^, CD4^+^ and CD4^+^/CD8^+^ counts were increased while CD8^+^ counts decreased in a dose dependent manner. Increasing dosage of PFVM resulted in increased levels of IL-2 and TNF-α. However, IL-2 levels were highest when a medium dose was administered while TNF-α was found to be highest when a high dose was administered. A separate study revealed that a water-soluble polysaccharide from *F. velutipes* (FVP l-A) resulted in increased NO and TNF-α production. IL-1β and IL-6 were also increased and lymphocyte proliferation was promoted (Wu et al., 2014). Similarly, the water extract of *F. velutipes* was also demonstrated to enhance splenocyte proliferation and Th1 cytokine production in mice (Ryu et al., 2014). Another interesting study by Lee et al. (2011) showed that the boiling water mushroom *F. velutipes* extract potentiated the production of IFNγ and cytotoxic activity against YAC-1 (lymphoma cell) of large intestinal lamina propria leukocytes but not small intestinal lamina propria leukocytes (Lee et al., 2011).

The human complement system also participates in the host defense system in both innate and adaptive immunity to protect the body from foreign invading agents such as bacteria, fungi and viruses (Carroll, 2004). Activation of the complement system can initiate a series of processes including opsonisation/phagocytosis, release of inflammatory mediators and the formation of the membrane attack complex, which subsequently leads to cell lysis. Under the normal physiological state, the effects of the complement system activation are beneficial to the host, but the excessive activation of the system may cause undesirable adverse effects which then contribute to the pathogenesis of autoimmune and inflammatory diseases. Therefore, the modulation of complement activity can be an important and useful tool in the effort to treat inflammatory diseases such as rheumatism and arthritis. Shin et al. (2007) investigated the anti-complement activity of seven different basidiomycetes extracts. The investigation revealed that both the hot water extracts and ethanol soluble fractions of *F. veluitipes* had the strongest anti-complement activity among the tested basidiomycetes extracts, with IC_50_ of inhibitory activity toward total hemolytic complement at 47.1% and 57.5%, respectively.

### Antimelanosis, anti-discoloration and anti-browning properties

There has been increased interest directed toward natural products with melanosis inhibiting properties because post-harvest melanosis occurring in seafood like crabs and shrimps reduces the potential value of the seafood thus affecting the economy (Encarnacion et al., 2011b). In addition, this work also has potential for developing natural whitening cosmetic products or natural food anti-browning product (Kim et al., 2011). Several experiments showed that ergothioneine **(34)** isolated from *F. velutipes* extract decreased polyphenoloxidase (PPO) activity which causes melanosis in different species of shrimps and crabs in a dose-dependent manner (Encarnacion et al., 2010, 2011a,b, 2012). Those studies also showed reduction in expression of prophenoloxidase (proPO) gene in the hemocytes of shrimps and crabs which had been submerged in ergothioneine-rich mushroom extract. Furthermore, the melanosis inhibition activity of the ergothioneine **(34)** from mushrooms was also demonstrated by the absence or reduction in blackening of the carapace of the treated shrimps and crabs compared to the untreated group. Encarnacion et al. (2012) also suggested that ergothioneine **(34)** may be a non-competitive inhibitor which possibly interacts directly with Cu^2+^ at the putative binding sites of polyphenol oxidase enzyme.

The extracts of several mushrooms containing ergothioneine **(34)** were also shown to prevent brown discoloration in processed fish meat (Bao et al., 2010b). Brown discoloration occurs when both deoxymyoglobin and oxymyoglobin are oxidized into metmyoglobin (metMb). The study suggested that *F. velutipes* extract is able to suppress the formation of metMB. Moreover, tyrosinase activity, which is involved in the production of melanin, was shown to be inhibited by ethyl acetate extract of *F. velutipes* mycelia grown in a specific medium containing 2% glucose (Kim et al., 2011). These studies have demonstrated the potential application of *F. velutipes* extract as an effective natural alternative to synthetic antimelanosis, anti-discoloration and anti-browning agents for the food industry.

### Antimicrobial properties: antibacterial and antifungal activities

Antimicrobial resistance is a significant global public health concern, particularly the emergence of multi-drugs resistant strains of pathogens which have developed resistance toward almost all the available antibiotics (Letchumanan et al., 2015a,b; Tan et al., 2016d). As a result, there is an increasing push to search for bioactive compounds from natural sources to serve as alternative antimicrobials (Tan et al., 2015b, 2016a; Azman et al., 2016; Chan et al., 2016) Reports on the occurrence of antimicrobials in *F. velutipes* mushroom are also well documented. The antimicrobial activities of extracts from different parts of *F. velutipes* have been the focus of several studies. Karaman et al. (2010) showed that both methanol and chloroform extracts from mature fruiting bodies of *F. velutipes* exhibited strong antibacterial activities, particularly against *Staphylococcus aureus* and *Bacillus subtilis*. Similarly, the methanol extract of Macedonian wild *F. velutipes* fruiting body was demonstrated to show antibacterial activity against both Gram-positive and Gram-negative bacteria, including *B. subtilis, Bacillus pumilus, S. aureus*, and *Pseudomonas aeruginosa* (Nedelkoska et al., 2013). Besides that, the antagonistic activity of *F. velutipes* against plant pathogenic fungi was also examined by evaluating the competitive interactions of the mushroom and the pathogens in a dual culture *in vitro* experiment (Borhani et al., 2011). The study showed that after an initial deadlock, *F. velutipes* was able to completely replace *Ophiostoma ulmi* by *F. velutipes*. *F. velutipes* also partially replaced the other three plant pathogenic fungi tested namely *Fusarium oxysporum, Pestalotiopsis funerea* and *Fusarium culmurum* LM2091 (Borhani et al., 2011). Recently, a study demonstrated that the extract from *F. velutipes* exhibited inhibitory activity toward the adhesion of pathogenic fungi (*Sporothrix schenckii* and *Candida albicans*) to epithelial cells (L929 cell line) (Kashina et al., 2016).

Enokipodins are a group of α-cuparene type sesquiterpenoids that have been isolated from *F. velutipes* and are known to be major constituents responsible for *F. velutipes* antimicrobial activities. Four enokipodins A **(35)**, B **(22)**, C **(36)** and D **(23)** with known chemical structures have been isolated and purified from the mycelial culture of *F. velutipes* by a group of researchers (Ishikawa et al., 2000, 2001). This group of compounds, enokipodins A–D **(22, 23, 35, 36)** were also successfully synthesized chemically (Srikrishna and Rao, 2004; Saito and Kuwahara, 2005; Secci et al., 2007). It was reported that enokipodins A-D **(22, 23, 35, 36)** exhibited antibacterial activity mainly against the Gram-positive bacteria such as *B. subtilis* and *S. aureus* (Ishikawa et al., 2001, 2005; Saito and Kuwahara, 2005). Moreover, both enokipodins A **(35)** and C **(36)** demonstrated minimum inhibitory doses against *B. subtilis* LMA0011 comparable to those of penicillin G (Ishikawa et al., 2005). A more recent study investigated the effect of culture conditions on the production and antimicrobial activity of the antimicrobial metabolites of *F. velutipes* (De Melo et al., 2009). The study revealed that dextrose potato broth best supported mycelia growth while complete Pontecorvo's culture medium resulted in greater antimicrobial metabolite production by *F. velutipes* (De Melo et al., 2009).

In addition to the discovery of enokipodins A–D **(22, 23, 35, 36)**, another research group from China isolated new cuparene-type sesquiterpenes from the solid culture of *F. velutipes* which also demonstrated antibacterial activities (Wang et al., 2012b). The chemical structures of these sesquiterpenes were elucidated and named as flamvelutpenoids A–D **(37** to **40)**. They were shown to exhibit antibacterial activity against *E. coli, B. subtilis* and methicillin-resistant *S. aureus* with MIC measuring more than 100 μM (Wang et al., 2012b). Furthermore, Wang et al. (2012d) also revealed the isolation of six new cuparene sesquiterpenes, enokipodins E–J **(25** to **30)** with antibacterial and antifungal activities. Enokipodins I **(29)** and J **(30)** were shown to exhibit antibacterial activity against *B. subtilis* with MICs of 164.3 ± 6.2 and 151.2 ± 4.5 μM, respectively. Meanwhile, enokipodins F, G and I **(26, 27, 29)** exhibited antifungal activity against *Aspergillus fumigatus* with MICs ranging between 229.1 and 235.1 μM. Interestingly, the antifungal potency of *F. velutipes* was found to be enhanced in selenium (Se)-enhanced cultivation medium (Milovanovic et al., 2015). The antifungal activity of the ethanol extract of *F. velutipes* mycelia supplemented with Se was shown to be enhanced, as evidenced by 8-fold lower MIC against *Candida parapsilosis* (Milovanovic et al., 2015).

Although numerous studies have demonstrated the antimicrobial potential of *F. velutipes* as well as the bioactive compounds responsible for the ascribed activity, additional work to understand their mechanisms of action would need to be undertaken before proceeding to practical implementation of any of these compounds as nutraceuticals or drugs in the food and pharmaceutical industries.

### Anti-inflammatory

Inflammation is known to be a complex biological response to infection and tissue injury that ultimately leads to recovery of tissue structure and function. However, prolonged inflammation contributes to the development of many inflammatory diseases (Supriady et al., 2015). Although many steroidal and nonsteroidal anti-inflammatory drugs have been introduced for anti-inflammatory therapy, their prolonged use has been reported to pose serious adverse effects including significant gastrointestinal upset, gastritis, renal problems, and even myocardial infarction and strokes (Hyllested et al., 2002; Basu and Hazra, 2006). Therefore, much interest has been shown toward alternative anti-inflammmatory agents of plant origin as they appear to be natural and safe drugs which pose minimal, if any, adverse effects (Supriady et al., 2015). A well-known medicinal mushroom, *F. velutipes* has been shown to possess anti-inflammatory activities. Gunawardena et al. (2014) revealed that unprocessed *F. velutipes* mushrooms possess anti-inflammatory properties, inhibiting the production of NO (IC_50_ = 0.024 ± 0.01 mg/mL) and TNF-α (IC_50_ = 0.099 ± 0.012 mg/mL) from murine macrophage RAW264.7 activated by lipopolysaccharides and IFN-γ. Additionally, the study showed that mushrooms that had undergone food processing steps such as boiling and heating showed less potent anti-inflammatory property, suggesting that the anti-inflammatory bioactive factors may have been degraded in the processed mushroom. Another study showed that the water and ethanol extracts of *F. velutipes* exhibited strong nitric oxide inhibitory activity and also inhibitory effect on iNOS and COX-2 expression in macrophages (Kang, 2012).

### Hepatoprotective activity

Recently, a considerable number of studies have focused on the characterization of polysaccharides of mushrooms due to their protective action against hepatotoxicity (Wu et al., 2011; Gan et al., 2012; Soares et al., 2013; Liu et al., 2014). *F. velutipes* is among the mushrooms documented to have potential in exerting protective effect toward hepatocytes. Pang et al. (2007), isolated an α-(1 → 4)-d-glucan, a water soluble polysaccharide (FVP2) with hepatoprotective activitiy from *F. velutipes*. The study indicated that FVP2 enhanced the growth of primary hepatocytes from mice *in vitro* significantly at concentrations ranging from 25 to 200 μg/mL. Pang et al. (2007) demonstrated the hepatoprotective effect of FVP2 was mediated by the inhibition of the release of intracellular alanine aminotransferase (ALT) from the intoxicated hepatocytes induced by carbon tetrachloride (CCl_4_). The authors also suggested that FVP2 may have protected the hepatocytes by preventing the production of CCl3^•^ radical caused by CCl_4_, subsequently inhibiting lipid peroxidation and intracellular release of ALT (Pang et al., 2007).

## Limitations and future works

The collective evidence presented in this review strongly suggests that *F. velutipes* should be exploited as a great source for development of functional foods, nutraceuticals and even pharmaceutical drugs. Despite that, there are still many challenges that need to be faced in order to facilitate the development of these natural products before they enter the pharmaceutical markets (Wasser, 2011). One of them is that considerable effort is required to perform precise identification of specific bioactive molecules responsible for the bioactivity of the mushroom extracts. This provides a better understanding of the mechanism of action of each particular compound on the ascribed bioactivity. Furthermore, this also would address the issue of whether the bioactive effects are caused by a single component or are the result of a synergistic effect from several components in the extracts.

There is also insufficient quality control and regulatory protocols to guarantee a standardized extraction process and the eventual quality and efficacy of mushroom-derived natural products. For instance, some compounds such as polysaccharides, are highly diversified in structure and molecular weight (Jing et al., 2014); hence, it is challenging to maintain batch to batch quality. Without consistency in the quality of the mushroom-derived natural products, the composition and the effectiveness of any commercially available preparation of mushroom products would be highly variable and different. Hence, only proper standards and protocols can ensure the product quality. Moreover, it is also important to establish simple and reliable analytic techniques to assess the authenticity or detect adulteration in mushrooms; preventing adverse effects attributed to adulteration. Apart from the use of gene markers to authenticate *F. velutipes* (Su et al., 2008; Zhang et al., 2010), a recent effort has shown that the analysis of IR spectroscopic fingerprints using principal component analysis is a reliable identification and qualification technique suitable for quality control of the polysaccharides extracted from *F. velutipes* (Jing et al., 2014).

Even with the current knowledge on *F. velutipes*, there is still limited work assessing the bioavailability, pharmacokinetics and pharmacodynamics of the bioactive compounds isolated from *F. velutipes*. A full understanding of the pharmacokinetic profiles of the natural products from these mushrooms is required as it impacts their bioactivity after metabolism. For instance, information regarding the ability of a bioactive compound to be absorbed from the site of administration and to pass through several biological barriers are crucial for the compound to exert its effect at the target site (Chen et al., 2011). To date, there have been abundant scientific investigations involving extracts or compounds of this mushroom and their potential health promoting benefits toward mankind, mainly on the basis of *in vitro* and *in vivo* animal trials. However, clinical studies exploring the therapeutic potential of *F. velutipes* are unfortunately very few in number. Therefore, clinical trials are highly needed to ascertain the dosage, efficacy and safety of these compounds as adequate alternatives to currently available drugs.

## Conclusion

An increasing awareness of the potential side effects of synthetic therapeutic agents and nutraceuticals has led to increased efforts to seek out natural products with beneficial effects on health. Studies have shown that *F. velutipes*, an easily available mushroom, has excellent nutritional value and also remarkable potential applications for medicinal purposes. Nutritionally, *F. velutipes* is a good source for carbohydrates, proteins, unsaturated fatty acids, many valuable micronutrients and dietary fiber which are highly comparable to vegetables. Although the nutritional values and culinary uses of *F. velutipes* are well documented, its medicinal qualities have yet to be. Bioactive polysaccharides extracted from *F. velutipes* have been demonstrated to possess a wide variety of bioactivities, particularly anticancer, immunomodulatory and anti-neurodegenerative effects. However, the exact mode of action of these carbohydrates is still elusive and deserves special attention in future study. Extensive research has also focused on a fungal immunomodulatory protein, FIP-fve isolated from *F. velutipes*, which exhibits both promising anticancer and immunomodulatory effects. The majority of the studies showed congruent findings that the FIP-fve elicits its immunomodulatory effects by shifting toward Th1 immune response. Apart from that, extracts of *F. velutipes* are also found to possess anticancer, anti-atherosclerotic, anti-thrombotic, cholesterol lowering, anti-hypertensive, memory and learning enhancement, antioxidant, antiaging, immunomodulatory, melanosis inhibition, anti-inflammatory, anti-complement, antimicrobial and hepatoprotective activities. A number of chemically defined bioactive molecules from diverse chemical groups have also been isolated from *F. velutipes* extracts that show invaluable prospects for future applications in the form of nutraceuticals/dietary supplements. In conclusion, *F. velutipes* has great potential as a nutraceutical and functional food, as well as potentially representing a valuable source for bioactive compounds therapeutic use and pharmaceutical application. The information presented in this review may also form the basis of providing adequate knowledge for future studies and developments as well as commercial exploitation of this fascinating mushroom.

## Author contributions

CT, PH, and LT contributed to the literature database search, data collection, data extraction and writing of the manuscript. LL, KC, PP, TK, and BG contributed vital insights and proofread the writing. The research topic was conceptualized by BG.

### Conflict of interest statement

The authors declare that the research was conducted in the absence of any commercial or financial relationships that could be construed as a potential conflict of interest.
